# Genome-wide survey and comprehensive expression profiling of Aux/IAA gene family in chickpea and soybean

**DOI:** 10.3389/fpls.2015.00918

**Published:** 2015-10-27

**Authors:** Vikash K. Singh, Mukesh Jain

**Affiliations:** Functional and Applied Genomics Laboratory, National Institute of Plant Genome ResearchNew Delhi, India

**Keywords:** gene family, *Aux/IAA*, chickpea, soybean, gene duplication, transposed duplication, gene expression, abiotic stress

## Abstract

Auxin plays a central role in many aspects of plant growth and development. *Auxin*/*Indole-3-Acetic Acid* (*Aux/IAA*) genes cooperate with several other components in the perception and signaling of plant hormone auxin. An investigation of chickpea and soybean genomes revealed 22 and 63 putative *Aux/IAA* genes, respectively. These genes were classified into six subfamilies on the basis of phylogenetic analysis. Among 63 soybean *Aux/IAA* genes, 57 (90.5%) were found to be duplicated via whole genome duplication (WGD)/segmental events. Transposed duplication played a significant role in tandem arrangements between the members of different subfamilies. Analysis of Ka/Ks ratio of duplicated *Aux/IAA* genes revealed purifying selection pressure with restricted functional divergence. Promoter sequence analysis revealed several *cis*-regulatory elements related to auxin, abscisic acid, desiccation, salt, seed, and endosperm, indicating their role in development and stress responses. Expression analysis of chickpea and soybean *Aux/IAA* genes in various tissues and stages of development demonstrated tissue/stage specific differential expression. In soybean, at least 16 paralog pairs, duplicated via WGD/segmental events, showed almost indistinguishable expression pattern, but eight pairs exhibited significantly diverse expression patterns. Under abiotic stress conditions, such as desiccation, salinity and/or cold, many *Aux/IAA* genes of chickpea and soybean revealed differential expression. qRT-PCR analysis confirmed the differential expression patterns of selected *Aux/IAA* genes in chickpea. The analyses presented here provide insights on putative roles of chickpea and soybean *Aux/IAA* genes and will facilitate elucidation of their precise functions during development and abiotic stress responses.

## Introduction

Auxin regulates cell division and elongation to drive plant growth and development (Woodward and Bartel, 2005). Perception of auxin and control of auxin-regulated gene expression is mediated by proteins belonging to three families including, receptors (F-box proteins), repressors [Auxin/Indole-3-Acetic Acids (Aux/IAAs)] and transcription activators auxin response factors (ARFs). The transmission of auxin signal depends upon interactions between components of these protein families. Under low concentration of auxin, formation of an ARF-Aux/IAA hetero-dimer results in the repression of target ARF transcription factors (Tiwari et al., 2001; Guilfoyle and Hagen, 2007). When auxin concentration is high, a co-receptor complex consisting of an F-box protein from the transport inhibitor response1 (TIR1)/auxin signaling F-box protein (AFB) family and an Aux/IAA protein, binds auxin (Dharmasiri et al., 2005; Tan et al., 2007). The F-box protein, being a component of a Skp1–Cullin–F-box (SCF) E3 ubiquitin ligase (Gray et al., 2001; Kepinski and Leyser, 2005), polyubiquitinylates and targets the Aux/IAA proteins for degradation (Maraschin et al., 2009). This degradation event relieves ARF transcription factor repression, thus allowing auxin-regulated gene transcription (Reed, 2001; Tiwari et al., 2001).

Aux/IAA proteins contain four conserved sequence motifs, among which motif I, an amino-terminal leucine repeat motif (LxLxLx) functions as transcriptional repressor of downstream auxin-regulated genes (Tiwari et al., 2001, 2004). Motif II, a TIR1/AFB recognition sequence with the conserved degron-sequence, GWPPV, is responsible for the stability of Aux/IAA proteins (Tiwari et al., 2004). Its interaction with the F-box protein, TIR1, leads to rapid degradation of Aux/IAA proteins (Dharmasiri et al., 2005). Motif III contains a βαα region and motif IV represents an acidic region (Hagen and Guilfoyle, 2002; Liscum and Reed, 2002). Motifs III and IV of Aux/IAA proteins enable homo- and/or hetero-dimerization with other Aux/IAA or ARF proteins and control the expression of downstream auxin-responsive genes (Kim et al., 1997; Ulmasov et al., 1997; Remington et al., 2004; Overvoorde et al., 2005). Although the presence of four conserved motifs is characteristic of Aux/IAA family, some members do not have one or more of these motifs and are called non-canonical members (Reed, 2001; Jain et al., 2006; Wang et al., 2010a; Audran-Delalande et al., 2012). Particularly, some members lack conserved motif II and are incapable of being recognized by TIR1/AFB proteins, indicating that these Aux/IAA proteins may be involved in other auxin-regulated biological processes (Jain et al., 2006; Kalluri et al., 2007; Sato and Yamamoto, 2008; Wang et al., 2010a,b; Audran-Delalande et al., 2012).

Many *Aux/IAA* genes have been characterized on the basis of mutant analysis in *Arabidopsis*, which demonstrated the important functions of Aux/IAA family genes in various developmental processes. For example, functional loss of IAA1/AXR5, a substrate of SCF^TIR1^, showed auxin-related growth defects and auxin-insensitive phenotype (Yang et al., 2004). Loss-of-function mutant, *iaa3/shy2*, affects auxin homeostasis and formation of lateral roots (Uberti-Manassero et al., 2012). The mutants, *iaa7/axr2*, *iaa17/axr3*, *iaa19/msg2*, and *iaa28* showed reduction in lateral root numbers (Tatematsu et al., 2004; Okushima et al., 2007; Uehara et al., 2008; Rinaldi et al., 2012), whereas *iaa14/slr* mutant blocked lateral root formation entirely (Fukaki et al., 2002). A gain-of-function mutant, *iaa16*, showed hampered plant growth and decreased response to auxin (Rinaldi et al., 2012). In rice, over-expression of *OsIAA1* led to inhibition of root elongation and shoot growth (Song et al., 2009) and a gain-of-function in *OsIAA11* resulted in the loss of lateral roots (Zhu et al., 2012). *OsIAA23* was found to be involved in post embryonic maintenance of quiescent center in rice (Jun et al., 2011).

The members of Aux/IAA gene family have been identified in several plant species, including *Arabidopsis* (Liscum and Reed, 2002), rice (Jain et al., 2006), *Populus* (Kalluri et al., 2007), maize (Wang et al., 2010b), tomato (Wu et al., 2012), *Vitis vinifera* (Cakir et al., 2013), and *Medicago* (Shen et al., 2014). However, a genome-wide analysis of Aux/IAA gene family in chickpea and soybean (for which genome sequences are available) is lacking as of now. Chickpea and soybean are very important legume crops, which serve as major source of proteins and carbohydrate. Considering diverse role of Aux/IAA family members in other plants, it is important to explore this gene family in chickpea and soybean. In this study, we identified *Aux/IAA* genes in chickpea and soybean genomes. We analyzed their sequence characteristics, genomic organization, *cis*-regulatory elements, and performed evolutionary duplication analysis. Furthermore, we analyzed spatio-temporal differential expression between Aux/IAA paralogs in various tissues/stages of development and under abiotic stress conditions. These data would facilitate future studies on elucidating the exact biological functions of *Aux/IAA* genes in legumes.

## Materials and Methods

### Identification of *Aux/IAA* Genes

Kabuli and desi chickpea genome annotations were downloaded from Legume Information System^1^ (LIS; Varshney et al., 2013) and Chickpea Genome Analysis Project^2^ (CGAP2; Parween et al., 2015), respectively. Soybean genome annotation was downloaded from Phytozome (v10, www.phytozome.net). Chickpea and soybean proteomes were searched to identify Aux/IAA proteins via HMMER and Basic Local Alignment Search Tool (BLASTP) algorithms using the published *Arabidopsis* Aux/IAA protein sequences as query. All obtained protein sequences were examined for the presence of Aux/IAA (PF02309) domain using the Hidden Markov Model of Pfam^3^ and SMART^4^ tools. Physiochemical parameters of each gene were calculated using ExPASy compute pI/Mw tool^5^. Information regarding cDNA sequences, genomic sequences and ORF lengths were obtained from the GFF file available at the respective genome project webpages.

### Gene Structure, Phylogenetic Analysis, and Motif Prediction

Analysis of exon/intron organization of the *Aux/IAA* genes was performed with Gene Structure Display Server^6^ (GSDS). Multiple sequence alignments of the full-length protein sequences from chickpea, soybean, and *Arabidopsis* were performed with MAFFT using default parameters and phylogenetic tree was constructed by UPGMA method using CLC Genomics Workbench (v4.7.2). Bootstrap analysis was performed using 1,000 replicates and the tree was viewed using FigTree (v1.3.1). Motif organization of chickpea and soybean Aux/IAA proteins was investigated by MEME web server^7^.

### Chromosomal Location and Gene Duplication

Information about the chromosome location was obtained from the GFF file and details of the segmentally duplicated regions in the soybean genome were retrieved using the SyMAP database (Soderlund et al., 2006). Synteny analysis for *GmIAA* genes was performed using Plant Genome Duplication Database^8^ (PGDD). The genes and segmental duplicated regions were mapped to the soybean chromosomes using the Circos tool (Krzywinski et al., 2009). On the basis of *K*s value obtained for each gene pairs from PGDD, divergence time was calculated to investigate evolution of soybean *Aux/IAA* genes. The divergence time (T) was calculated as *T* = Ks/(2 × 6.1 × 10^-9^) × 10^-6^ Mya, based on a rate of 6.1 × 10^-9^ substitutions per site per year. For Ks value less than 0.3, divergence time was after the *Glycine* whole genome duplication (WGD) event, when Ks value was more than 1.3, divergence time was after the gamma WGT (whole genome triplication) event, and if Ks value was between 0.3 and 1.3, divergence time was after legume WGD event and before the *Glycine* WGD event. To determine the significance or contribution of the transposed duplication in *Aux/IAA* gene evolution, Soytedb^9^ was investigated to find out nearest transposable elements around *Aux/IAA* genes.

### Expression Profiling Using RNA-seq and Microarray Data

For expression profiling in chickpea, we used the RNA-seq data of 17 different tissues, namely germinating seedling (GS), root (R), shoot (S), stem (ST), mature leaves (ML), young leaves (YL), shoot apical meristem (SAM), flower bud stages (FB1-4), flower stages (FL1-5), and young pod (YP) from previous studies (Jain et al., 2013; Singh et al., 2013). High quality filtered reads were mapped to the genome sequence of kabuli chickpea (Varshney et al., 2013) using TopHat (v2.0.6). Cuﬄinks tool was used to estimate the transcript abundance of genes in the form of fragments per kilobase of transcript per million reads (FPKM) in different tissues as described previously (Garg et al., 2015).

The expression of 63 *GmIAA* genes was investigated based on the RNA-seq data from 19 tissues available at Gene Expression Omnibus (GEO) database, including three samples from soybean seed compartments, GloEP (globular stage embryo proper; GSM721717), EmSCP (early maturation seed coat parenchyma; GSM721719), and GloS (globular stage suspensor; GSM721718); 10 other tissues samples, Gs (globular stage seed; GSM721725), Hs (heart stage seed; GSM721726), Cs (cotyledon stage seed; GSM721727), Es (early maturation stage seed; GSM721728), Ds (dry seed; GSM721729), R (root; GSM721731), ST (stem; GSM721732), L (trifoliate leave; GSM721730), FB (floral bud; GSM721733), and WS (whole seedling 6 days after imbibition; GSM721734); three cotyledon development samples, CoM (mid-maturation cotyledon; GSM721277), CoL (late maturation cotyledon; GSM721278), and CoS (seedling cotyledon; GSM721280); and three early maturation seed parts, EcoEm (early maturation embryonic cotyledon; GSM1213 856), EmEA (early maturation embryonic axis; GSM1213857), and EmSC (early maturation seed coat; GSM1213855). For the expression analysis of *GmIAA* genes, the RPKM method was employed to correct for biases in total gene size and normalize for total reads obtained in each tissue library (Mortazavi et al., 2008; Nagalakshmi et al., 2008). Heatmaps of normalized expression values of *Aux/IAA* genes of chickpea and soybean were generated using R package pheatmap.

For abiotic stress response analysis of chickpea *Aux/IAA* genes, we used raw RNA-seq data from root and shoot under desiccation, salt and cold stresses from our previous study (Garg et al., 2015). Read mapping and differential gene expression analysis was performed as described (Garg et al., 2015) using the kabuli chickpea genome as reference. The microarray data of soybean under salt and drought stresses were downloaded from the GEO database from accession numbers GSE41125 and GSE40627, respectively. Probe sets corresponding to the *GmIAA* genes were identified from the file GeneModels_AffyProbe.txt^10^.

### Plant Materials, RNA Isolation and Quantitative PCR Analysis

Chickpea (*Cicer arietinum* L. genotype ICC4958) seeds were grown in field and culture room for collection of various tissue samples. From field grown plants, mature leaf (ML), young leaf (YL), flower buds (FB1-FB4; where FB1, FB2, FB3, and FB4 were 4, 6, 8, and 8–10 mm size flower buds, respectively), flowers (FL1–FL5; where FL1 was young flower with closed petals, FL2 was flower with partially opened petals, FL3 was mature flower with fully opened petals, FL4 was mature flower with opened and faded petals and FL5 was drooped flower with senescing petals), young pods (YP) were harvested as described (Singh et al., 2013). Root (R), shoot (S), and GSs were harvested as described (Garg et al., 2010; Singh et al., 2013). Abiotic stress treatments (desiccation, salinity, and cold) were given and root and shoot tissue were harvested as described (Garg et al., 2010, 2015). Total RNA was isolated, quality, and quantity were checked as described (Singh et al., 2015). Gene-specific primers for selected *CaIAA* genes were designed using the Primer Express (v3.0) software (Applied Biosystems, Foster City, CA, USA) (Supplementary Table S1). Specificity of each primer pair was determined via BLAST search. Quantitative PCR reactions for at least two biological replicates each with three technical replicates were performed employing 7500 fast real-time PCR system (Applied Biosystems) as previously described (Garg et al., 2010). *Elongation factor-1 alpha* (*EF-1α*) was used as a reference gene for normalization of gene expression levels (Garg et al., 2010). Statistical significance of the differential expression patterns was determined using the Student’s *t*-test. Genes with ≥ 2-fold expression change (in at least one tissue/condition/time point) with *P* ≤ 0.05 were regarded as differentially expressed.

### Promoter Sequence Analysis

Genomic co-ordinates of coding sequences were determined using GFF files obtained from chickpea and soybean genome annotation projects. The regions of 1,000 bp upstream from start codon were extracted from the genome sequences. *Cis*-regulatory elements on both strands of promoter sequences were scanned using NewPLACE web server^11^.

## Results and Discussion

### Identification of *Aux/IAA* Genes in Chickpea and Soybean

In order to identify the members of *Aux/IAA* gene family in chickpea (kabuli) and soybean genome, BLASTP and HMM profile searches were performed against their respective proteomes. The *Aux/IAA* gene family members identified via these two searches were combined and a non-redundant list was obtained for chickpea and soybean. For further confirmation and identification of the conserved Aux/IAA domains, all candidate proteins were subjected to domain analysis using Pfam and SMART databases. A total of 22 and 63 Aux*/IAA* genes in kabuli chickpea and soybean genome, respectively, were confirmed. Further the analysis of recent version of desi chickpea genome (CGAP2) identified 21 Aux/IAA family members. BLASTP analysis showed the presence of all these 21 *Aux/IAA* genes in the kabuli chickpea genome. Due to identification of higher number of genes, all further analyses were performed on *Aux/IAA* genes from kabuli chickpea. Chickpea and soybean genes were numbered according to their location on the chromosomes (Supplementary Table S2). Various information of *CaIAA* and *GmIAA* genes, including gene name, gene identifier, chromosome location, mRNA length, features of deduced protein sequences, and their gene, CDS, protein and promoter sequences are given in Supplementary Table S2.

The number of *CaIAA* members (22) identified in chickpea are less as compared to *Arabidopsis* (29; Liscum and Reed, 2002) and rice (31; Jain et al., 2006), but higher than its very close relative *Medicago* (17; Shen et al., 2014). Lesser number of *Aux/IAA* genes in chickpea and *Medicago* may be due to some evolutionary constraints. However, the number of *Aux/IAA* members in soybean (63) is much higher as compared to other plants. Soybean possesses 9.2-fold larger genome size (∼1,150 Mbp) and 1.75-fold higher gene count (∼46,400) than *Arabidopsis* (Cannon and Shoemaker, 2012). Given the noticeable differences in genome size and estimated gene count between soybean and *Arabidopsis*, the *Aux/IAA* genes in soybean seem to be highly expanded. The presence of twice as many of these genes in soybean versus *Arabidopsis* may be mainly due to the recent polyploidy and segmental duplication events in soybean evolutionary history (Schmutz et al., 2010). The sizes of the CaIAA proteins varied markedly ranging from 112 (CaIAA16) to 362 (CaIAA2) amino acids. Similarly, sizes of GmIAA proteins also varied from 53 (GmIAA6) to 367 (GmIAA48) amino acids. Furthermore, predicted isoelectric points varied from 4.64 (CaIAA19) to 9.63 (CaIAA1) in chickpea and 5.24 (GmIAA42) to 9.27 (GmIAA25) in soybean, suggesting that different CaIAA and GmIAA proteins might function in different microenvironments.

### Phylogenetic Relationship, Gene Structure and Sequence Similarity

**FIGURE 1:**
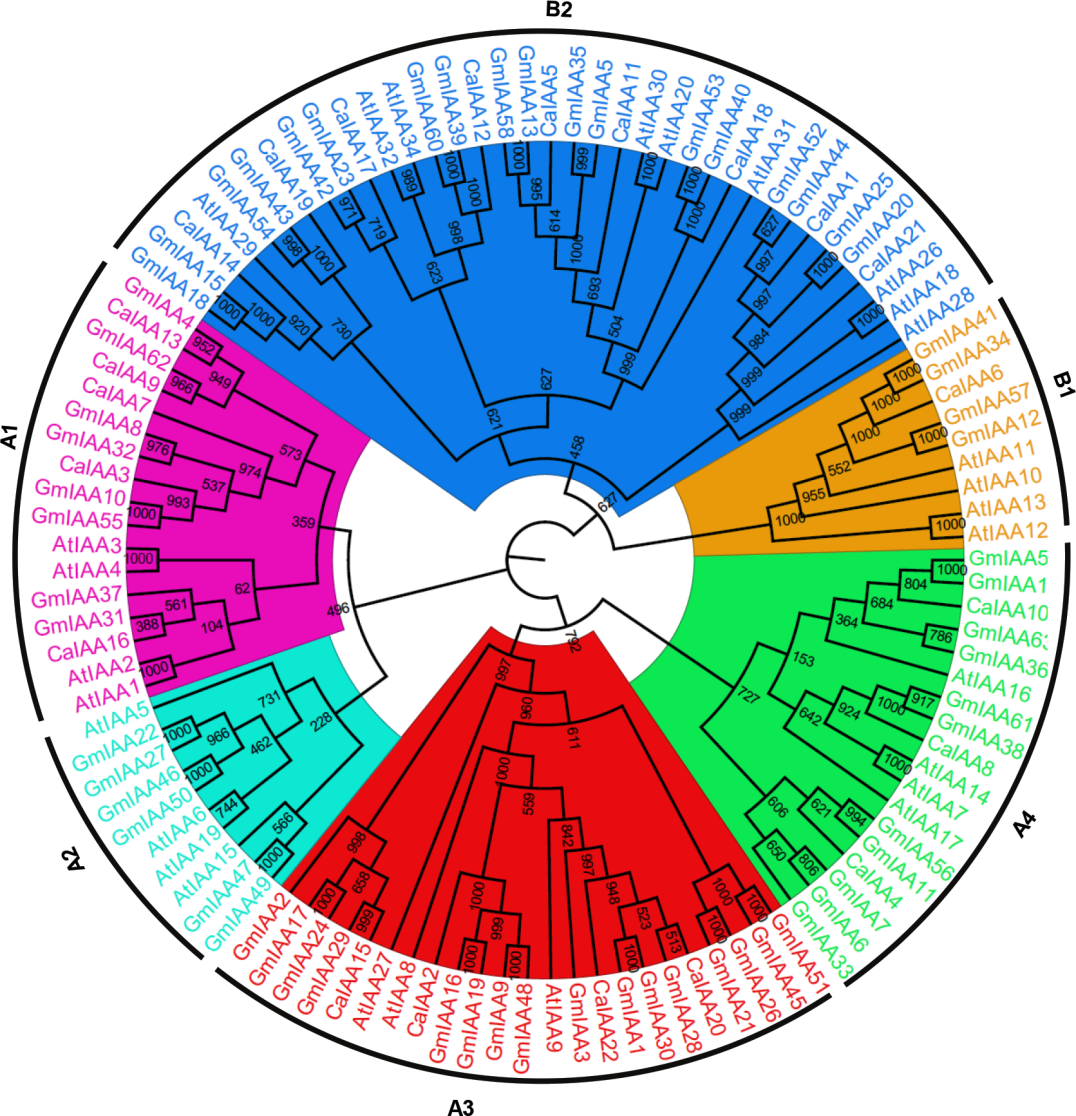
**Phylogenetic relationship among *Aux/IAA* genes from chickpea, soybean, and *Arabidopsis***. The deduced full-length amino acid sequences of chickpea (CaIAA), soybean (GmIAA), and *Arabidopsis* (AtIAA) genes were aligned by MAFFT and the phylogenetic tree was constructed by CLC Genomics Workbench using the UPGMA method. The members of each Aux/IAA subfamily are shown in different colors. The numbers on the nodes represent bootstrap values from 1000 replicates.

To examine the phylogenetic relationship among the Aux/IAA proteins of chickpea, soybean, and *Arabidopsis*, a rooted tree was constructed using alignments of their full-length amino-acid sequences (**Figure 1**). Phylogenetic distribution indicated that Aux/IAA proteins can be classified into two major groups, A and B (**Figure 1**) similar to *Arabidopsis* and rice (Remington et al., 2004; Jain et al., 2006), which are further subdivided into four and two subgroups, respectively. Similar groupings have been reported in other plant species too (Cakir et al., 2013; Gan et al., 2013). The group A (A1–A4) consisted of 12 members of CaIAA and 41 GmIAA proteins, structuring 25 sister pairs (five pairs of GmIAA-CaIAA, 16 pairs of GmIAA-GmIAA and four pairs of AtIAA-AtIAA proteins). Group B (B1–B2) included 10 CaIAA and 22 GmIAA proteins, which formed 15 sister pairs (11 pairs of GmIAA–GmIAA, four pairs of AtIAA–AtIAA). Phylogenetic tree topology revealed that sister pairs located at the terminal nodes show high similarity and were assigned as paralog or ortholog pairs (**Figure 1**, Supplementary Figure S1). The sequence similarity within chickpea and soybean Aux/IAA proteins ranged from 9 to 80.3 and 6.5 to 93.9%, respectively (Supplementary Figure S1). All paralog pairs of soybean determined through phylogenetic analysis were found to be duplicated via WGD events (**Figure 1**, Supplementary Table S3), except GmIAA6 and 7 (tandemly duplicated). Furthermore, higher sequence similarity was observed between paralog pairs, suggesting that these genes evolved via genome duplication event and may perform similar functions. Interestingly, phylogenetic analysis predicted four homologs of AtIAA16 in the soybean genome (GmIAA14, 36, 59, and 63). In *Populus*, four orthologs of AtIAA16 have also been found, but were absent in rice, indicating their specific function in dicots. Moreover, diversity of gene structure (exon-intron organization) is also a possible explanation for the evolution of multigene families. The exon-intron organization in the coding sequences of each *Aux/IAA* genes of chickpea and soybean were compared. As expected, in most of the sister-pairs, similar exon-intron organization was observed. This conservation of exon-intron organization between subfamilies and the dissimilarity within subfamilies supported the results of phylogenetic and genome duplication analysis.

**FIGURE 2:**
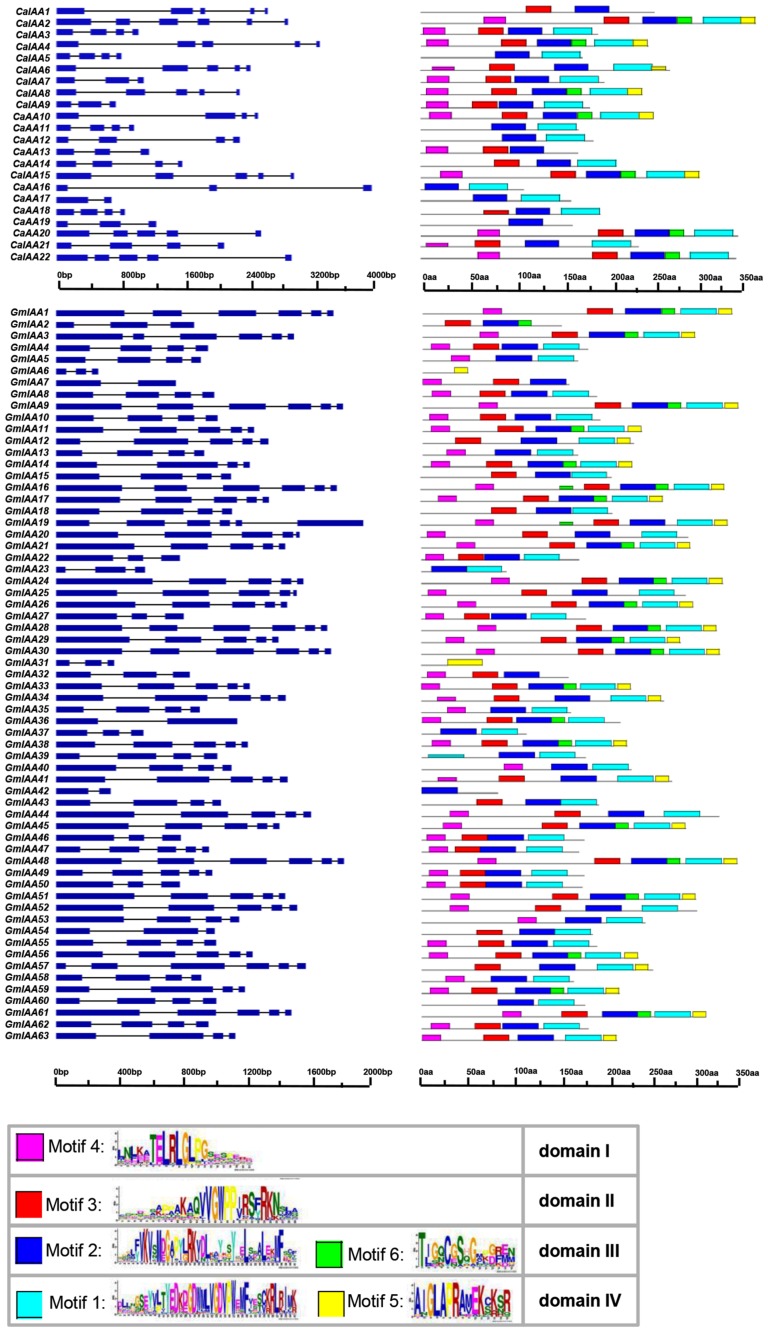
**Gene structure and motif organization of Aux/IAA family members in chickpea and soybean**. *Left panel* illustrates the exon–intron organization of *Aux/IAA* genes in chickpea and soybean. The exons and introns are represented by boxes and lines, respectively. *Right panel* shows motif organization in chickpea and soybean Aux/IAA proteins. Motifs of Aux/IAA proteins were investigated by MEME web server. Six motifs representing four domains I, II, III, and IV of Aux/IAA proteins are displayed at the bottom.

The established model for auxin signal transduction represents auxin-mediated degradation of these short-lived proteins that have four characteristic conserved domains. Conspicuously, the chickpea genome represents six such non-canonical Aux/IAA proteins (CaIAA5, 11, 12, 16, 17, and 19) that do not have conserved domain II, which is crucial for protein degradation, whereas 13 (GmIAA5, 6, 13, 23, 31, 35, 37, 39, 40, 42, 53, 58, and 60) such proteins were found in the soybean genome (**Figure 2**). These non-canonical proteins were found to be long-lived as compared to the canonical Aux/IAA proteins (Dreher et al., 2006). In tomato, such non-canonical Aux/IAA proteins were found to have expression pattern restricted to narrow development stages (Audran-Delalande et al., 2012), suggesting that these proteins may have a very specific function during development in plants for mediating auxin responses.

### Chromosomal Location and Duplication

**FIGURE 3:**
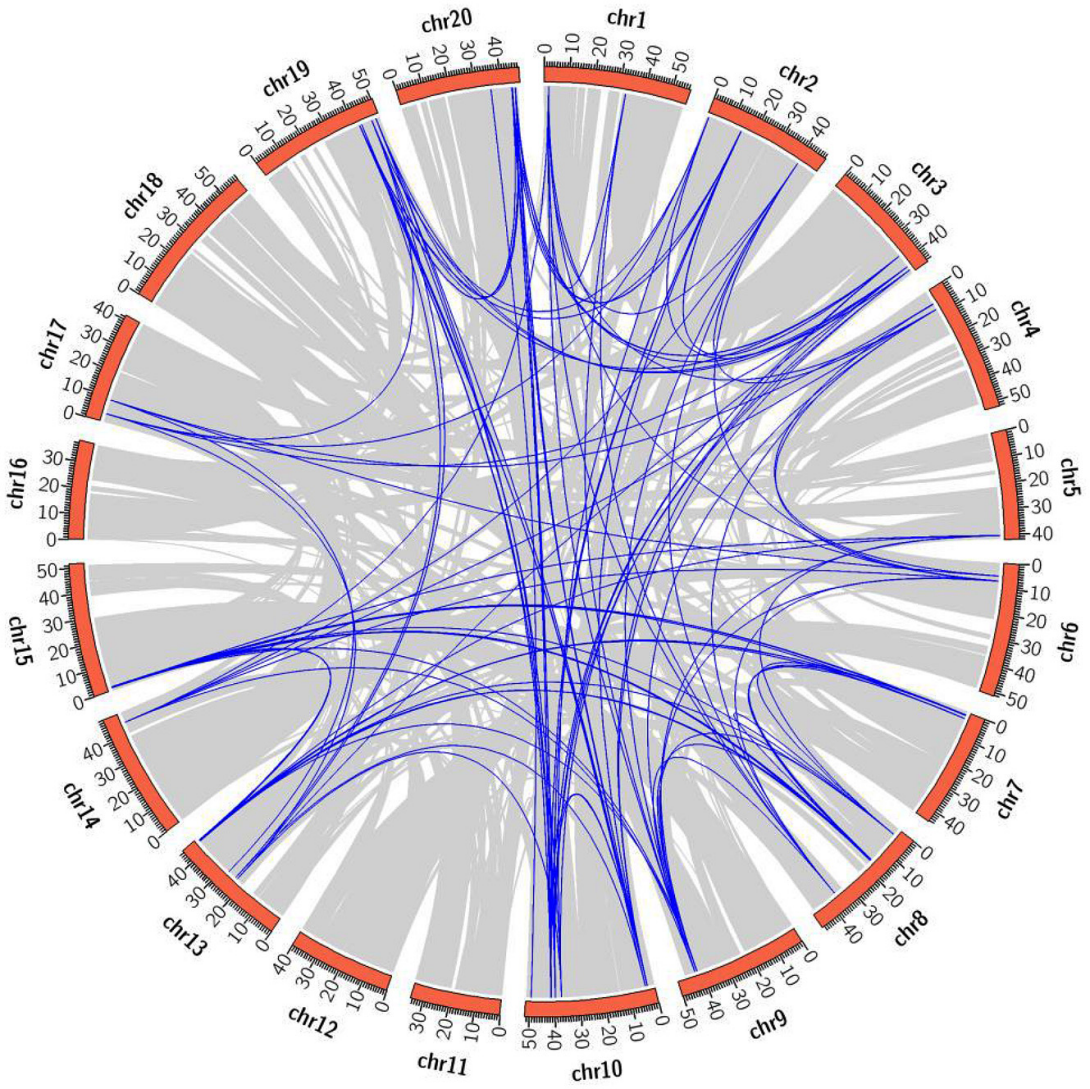
**Mapping of segmentally duplicated *GmIAA* genes on soybean chromosomes**. Gray ribbons indicate collinear relationships among the blocks in whole genome and blue ribbons show *GmIAA* paralogs. The soybean chromosomes are arranged in a red circle and the size of each arc show the size of respective chromosome (Mb).

The chromosomal distribution of 22 *CaIAA* genes revealed their location on all the eight linkage groups (Supplementary Table S2). Eight *CaIAAs* were present on chromosome 4, five on chromosome 7, three on chromosome 3, two on chromosome 6, and one on chromosome 1, 2, 5, and 8 each. In soybean, 63 *GmIAA* genes were located on 16 of 20 chromosomes, except for chromosomes 11, 12, 16, and 18 (**Figure 3**, Supplementary Table S2). Out of 63 *GmIAA* genes, nine genes were present on chromosome 10, eight on chromosome 13, six on chromosome 2, five on chromosome 3, 8, and 19 each, four on chromosome 7, 15, and 20 each, three on chromosome 1, and two on chromosome 4, 6, 9, and 17 each. Chromosomes 5 and 14 harbored only one *GmIAA* gene each. The chromosomal location of *Aux/IAA* genes of chickpea and soybean showed tandemly located gene clusters. The gene cluster in chickpea included *CaIAA3* and *4* on chromosome 3. For soybean, eight such clusters were observed, including *GmIAA6*, *7* and *8* on chromosome 2, *GmIAA10*, and *11* on chromosome 3, *GmIAA32* and *33*, *GmIAA37* and *38* on chromosome 10, *GmIAA46* and *47* on chromosome 13, *GmIAA49* and *50* on chromosome 15, *GmIAA55* and *56* on chromosome 19, and *GmIAA61* and *62* on chromosome 20.

Soybean genome has undergone one WGT and two WGD events (legume WGD and *Glycine* WGD), and about 75% genes have multiple paralogs (Schmutz et al., 2010; Severin et al., 2011). Among paralog genes, ∼50% displayed expression subfunctionalization (Roulin et al., 2012) that may cause phenotypic variation in polyploids (Buggs et al., 2010). Besides WGD, tandem duplication generates consecutive gene copies in the genome through unequal chromosomal crossing over (Freeling, 2009) and may contribute in the expansion of gene families (Cannon et al., 2004). Dispersed duplicates (not tandemly or segmentally duplicated) arises via either DNA or RNA based transposition mechanisms (Ganko et al., 2007; Cusack and Wolfe, 2007; Freeling, 2009) and may play an important role in altering gene function and creating new genes (Woodhouse et al., 2010; Wang et al., 2011). To find the potential relationship between putative paralog pairs of *Aux/IAA* genes of soybean and tandem/segmental duplications, we performed duplication analysis using PGDD. Within the identified duplicated *GmIAAs*, a larger fraction of them (57, 90.47%) were duplicated through WGD/segmental events, and only *GmIAA6* and *7* were tandemly duplicated (**Figure 3**, Supplementary Table S3). In the syntenic block, some genes from different subfamilies showed the tandem relationship. For example, paralog gene pairs, *GmIAA6/33*, *GmIAA10/55*, *GmIAA37/62*, and *GmIAA46/50* displayed tandem relationship with *GmIAA8/32*, *GmIAA11/56*, *GmIAA38/61*, and *GmIAA47/49*, respectively (**Figure 3**, Supplementary Tables S2 and S3). Presence of transposable elements in the flanking regions of these genes, suggested that they were tandemly arranged due to transposed duplication events (Supplementary Table S4). In addition to WGD events, some other gene duplication events were also found in few *Aux/IAA* genes of soybean. In A1 subfamily, six paralog genes (*GmIAA8*/*32*, *10*/*55*, and *37*/*62*) resulted from the common ancestor sites experiencing three WGD events, while a dispersed gene (*GmIAA4*) through the transposed duplication located among two transposable elements (Supplementary Tables S3 and S4). In A4 subfamily, ten paralog genes (*GmIAA6*/*33*, *11*/*56*, *14*/*59*, *36*/*63*, and *38*/*61*) were resultant from common ancestor sites, which experienced three WGD events, while *GmIAA7* was found to have tandem connection with *GmIAA6* (Supplementary Tables S3 and S4). The *GmIAA* genes grouped into B1 and B2 subfamilies exhibited transposed duplication, which were flanked by two transposable elements each (Supplementary Tables S3 and S4).

Furthermore, to investigate whether Darwinian positive selection is involved in the divergence of *GmIAA* genes after duplication and to trace the dates of the duplication blocks, the substitution rate ratios (Ka/Ks) of all paralog pairs were extracted from PGDD database. *K*s values were used for calculating approximate dates of duplication events. The segmental duplications of the *GmIAA* genes in soybean were assumed to originate from 2.35 Mya (million years ago, *K*s = 0.03) to 327 Mya (*K*s = 4.26), with a mean value of 17.6 Mya (*K*s = 0.23, Supplementary Table S5). Previous studies have shown that the soybean genome has undergone two rounds of WGD, including an ancient duplication prior to the divergence of papilionoid (58–60 Mya) and a *Glycine*-specific duplication that has been estimated to have occurred ∼13 Mya (Schmutz et al., 2010). Most of the WGD/segmental duplications of the *GmIAA* genes seem to have occurred around 13 Mya when *Glycine*-specific duplication occurred (Supplementary Table S5). According to the ratio of non-synonymous to synonymous substitutions (Ka/Ks), the history of selection acting on coding sequences can be measured (Li et al., 1981). A pair of sequences will have Ka/Ks <1, if one sequence has been under purifying selection, but the other has been drifting neutrally, while Ka/Ks = 1, if both the sequences are drifting neutrally and rarely, while Ka/Ks >1 at specific sites, when they were under positive selection (Juretic et al., 2005). Ka/Ks for all *GmIAA* duplicated pairs were less than 1 (Supplementary Table S3), which suggests that all gene pairs have evolved mainly under the influence of purifying selection pressure with limited functional divergence after segmental duplications.

### *Cis*-regulatory Elements in Promoters of *CaIAAs* and *GmIAAs*

The analysis of *cis*-regulatory elements in the promoter sequences is an important aspect in understanding the gene function and regulation. We searched 1 kb promoter region of all the *CaIAA* and *GmIAA* genes to determine putative *cis*-regulatory elements involved in their transcriptional regulation using NewPLACE database. Many *cis*-regulatory elements identified in the promoters were found to be related to auxin, ABA, SA, sugar, light, drought, salt, and cold responses indicating that these genes are linked to phytohormone signals, and/or abiotic stresses (Supplementary Table S6). Previous studies suggest that light is involved in regulation of Aux/IAA protein activity. For example, phytochrome A (phyA) interacts with Aux/IAA proteins, as revealed by yeast two-hybrid analysis (Soh et al., 1999). Moreover, oat phyA was able to phosphorylate IAA1, SHY2/IAA3, IAA9, AXR3/IAA17, and PS-IAA in vitro (Abel et al., 1994; Soh et al., 1999). The domain II mutants, *axr2-1*, *axr3-1*, *shy2-1*, and *shy2-2*, develop leaves in dark (Kim et al., 1998; Reed et al., 1998; Nagpal et al., 2000). Presence of auxin, ABA and cytokinin responsive *cis*-regulatory elements in the promoters of *CaIAA* and *GmIAA* is also consistent with previous reports, such as the interactions of auxin with other phytohormones (cytokinin or ABA) to regulate many aspects of plant growth and development (Paponov et al., 2008). Many *CaIAA* and *GmIAA* genes were found to harbor AuxRE motif in their promoter, which is important for binding of ARFs and transcriptional activation of *Aux/IAA* genes (Tiwari et al., 2001, 2003). Interestingly, promoter sequences of those *CaIAA* and *GmIAA* genes, which lack AuxRE motif, were found to harbor sugar-responsive motif (SREATMSD), suggesting that there is an association between sugar and auxin responses. Both sugar and auxin are essential for plants and control similar processes. In *Arabidopsis*, these two signaling pathways were found to interact with each other (Mishra et al., 2009). Bioactive GAs (gibberellic acid) influence nearly all aspects of plant growth and development from germination to hypocotyl elongation, stem growth, circadian rhythm, and reproductive organ and seed development (Lovegrove and Hooley, 2000; Hartweck and Olszewski, 2006). The presence of gibberellic acid response element (GARE) in many of the *Aux/IAA* genes (Supplementary Table S6), indicate their role in such processes. In addition, *cis*-regulatory elements known for regulation of endosperm, embryo, cotyledon, seed storage proteins related responses were also predicted in the *CaIAA* and *GmIAA* gene promoters (Supplementary Table S6), suggesting their role in seed development. Circadian element, which is involved in circadian control, was abundantly found in the promoter region of chickpea and soybean *Aux/IAA* genes (Supplementary Table S6), potentially indicating that they may have a distinct diurnal expression pattern. In *Arabidopsis* and rice, a class of element defined by the core motif ‘(a/g)CCGAC’ named as dehydration responsive element/C-repeat (DRE/CRT) was reported for drought, low temperature, and salt inducible expression (Yamaguchi-Shinozaki and Shinozaki, 1994; Meier et al., 2008). Interestingly, we also found this motif in many of the *CaIAA* and *GmIAA* gene promoters (Supplementary Table S6), indicating their role under abiotic stress conditions. Overall, the promoter analysis demonstrated the presence of a variety of *cis*-regulatory elements in the upstream regions of chickpea and soybean *Aux/IAA* genes. These results provide further support for the various functional roles of *Aux/IAA* genes in a wide range of developmental processes and abiotic stress responses.

### Differential Expression of Chickpea and Soybean Aux*/IAA* Genes during Development

**FIGURE 4:**
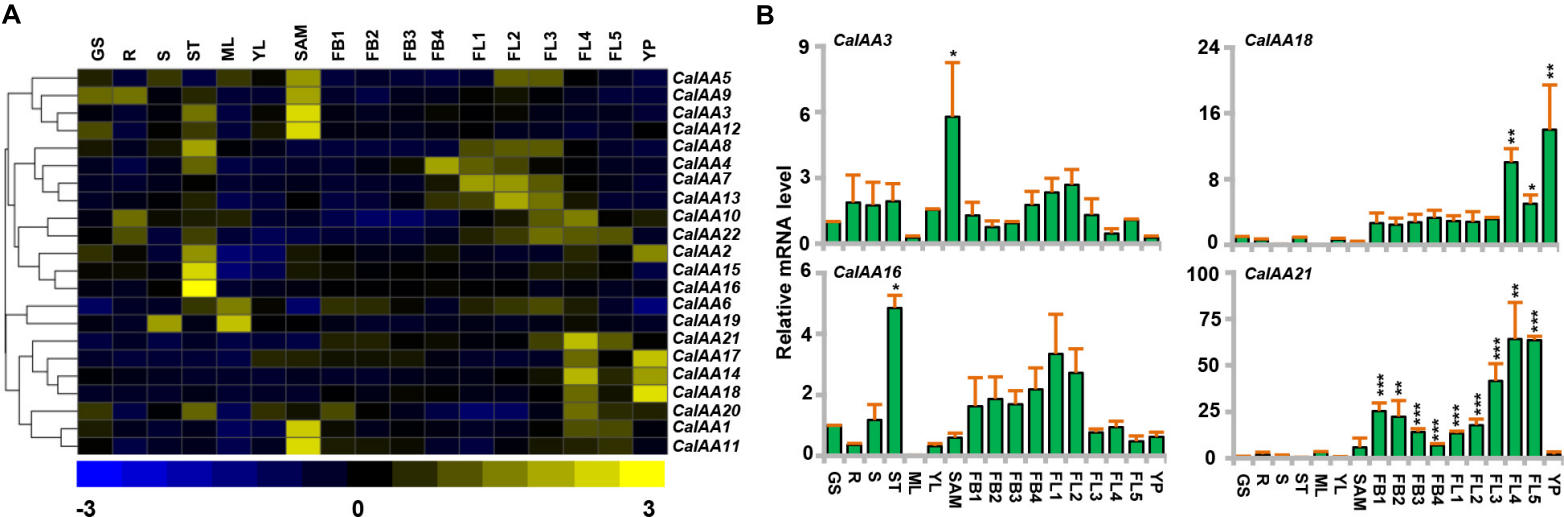
**Expression profiles of chickpea *Aux/IAA* genes**. **(A)** The normalized RNA-seq expression data was used to construct heatmap. Hierarchical clustering was conducted in R using the pheatmap package with a dissimilarity metrics based on Euclidean distance with complete linkage algorithm. Color key at the bottom represents row wise *Z*-score. **(B)** qRT–PCR analysis of *CaIAA* genes in various tissue/stages of development. Expression of germinating seedling (GS) was taken as a reference to determine relative mRNA level in other tissues for each gene. Error bars indicate SE of mean. R (root), S (shoot), ST (stem) ML (mature leaf), SAM (shoot apical meristem), FB1-4 (stages of flower bud), FL1-5 (stages of flower), YP (young pod). Data points marked with asterisk (^∗^*P* ≤ 0.05, ^∗∗^*P* ≤ 0.01, and ^∗∗∗^*P* ≤ 0.001) indicates statistically significant difference between control (GS) and other tissues.

To know the putative function of *Aux/IAA* genes in chickpea and soybean during development, we analyzed their expression profiles in different vegetative and reproductive tissues, using available RNA-seq data sets (Jain et al., 2013; Singh et al., 2013). Chickpea RNA-seq data included eight tissues/organs, such as GS, root (R), shoot (S), stem (ST), mature leaf (ML), young leaf (YL), SAM, young pod (YP) and nine stages of flower development (FB1-4 and FL1-5). Many of *CaIAAs* illustrated a distinct tissue-specific expression pattern (**Figures 4A,B**). For example, *CaIAA15* and *16* revealed specific expression in stem, indicating their role in stem development (**Figures 4A,B**; Supplementary Table S7). It has been found that mutation in *Aux/IAA* genes affect stem elongation (Reed, 2001). Furthermore, *CaIAA1, 3, 11*, and *12* showed higher expression in SAM, among which *CaIAA3* was validated through qRT-PCR (**Figures 4A,B**; Supplementary Table S7), implying their involvement in SAM maintenance. In rice, *OsIAA23* was found to be involved in postembryonic maintenance of quiescent center (Jun et al., 2011). *CaIAA4, 7, 10, 13*, and *21* revealed higher transcript accumulation during stages of flower development (**Figures 4A,B**; Supplementary Table S7), suggesting their possible role in flower development. In rice, *OsIAA4* and *26* were found to be up-regulated in panicle (Jain and Khurana, 2009), while *MtIAA9* in *Medicago*, showed higher expression level in flower (Shen et al., 2014). *CaIAA14, 17* and *18* revealed distinctly higher expression in stages of flower development and young pod (YP). In tomato, *SlIAA9* was shown to be involved in fruit development (Wang et al., 2005). The expression profile of at least four *CaIAA* (*CaIAA3*, *16*, *18*, and *21*) genes was studied by qRT-PCR to validate the RNA-seq results. The expression patterns obtained via qRT-PCR were found to be well correlated with that of RNA-seq (**Figures 4A,B**).

**FIGURE 5:**
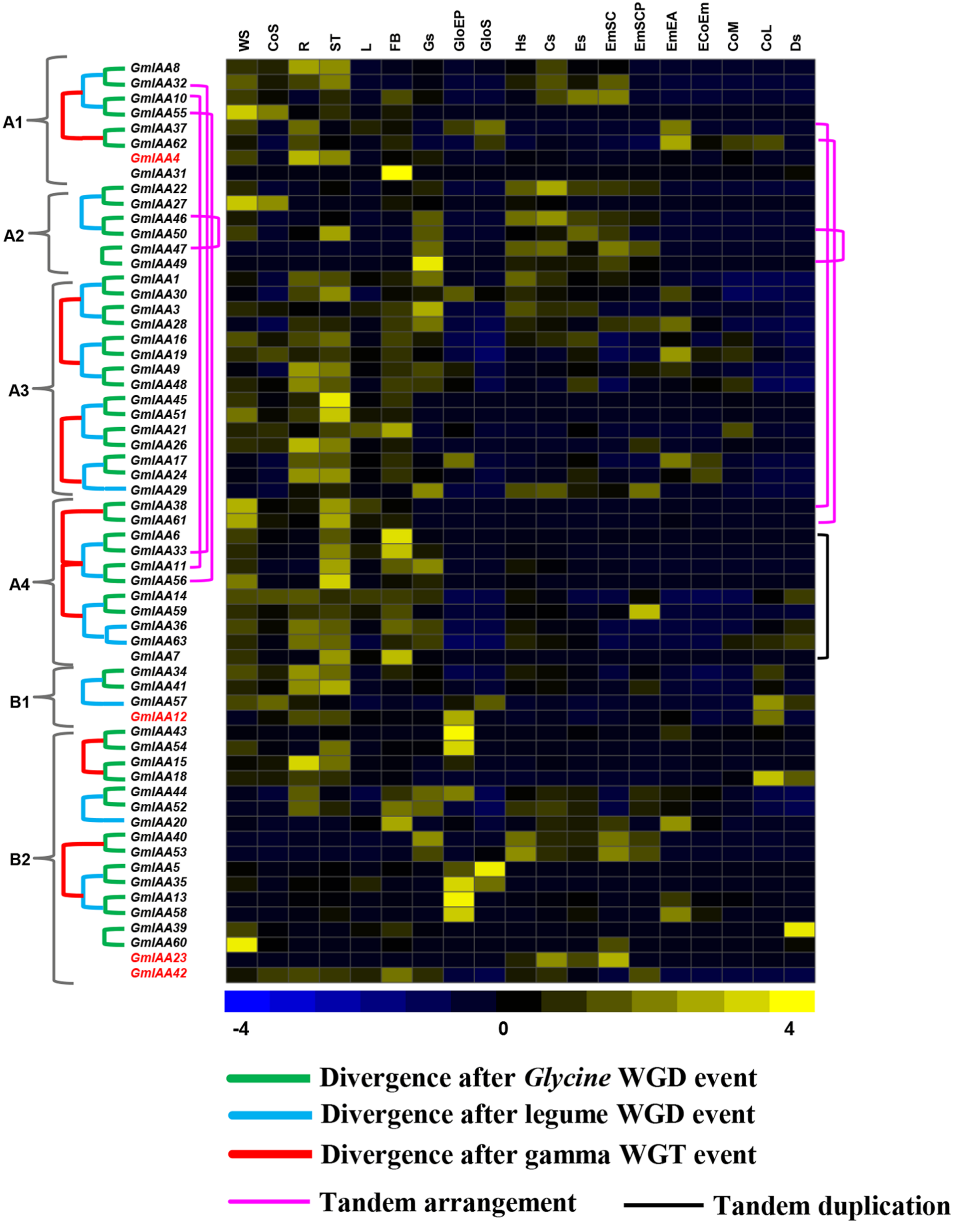
**Expression profiles and the evolutionary patterns of soybean *Aux*/*IAA* genes**. The normalized RNA-seq expression data of 19 tissues was used to construct heatmap. Three samples from soybean seed compartments: GloEP (globular stage embryo proper), EmSCP (early maturation seed coat parenchyma), and GloS (globular stage suspensor); 10 other tissues samples: Gs (globular stage seed), Hs (heart stage seed), Cs (cotyledon stage seed), Es (early maturation stage seed), Ds (dry Seed), R (root), ST (stem), L (trifoliate leave), FB (floral bud), and WS (whole seedling 6 days after imbibition); three cotyledon development samples: CoM (mid-maturation cotyledon), CoL (late maturation cotyledon), and CoS (seedling cotyledon); three early maturation seed parts: EcoEm (early maturation embryonic cotyledon), EmEA (early maturation embryonic axis), and EmSC (early maturation seed coat). The lines show the syntenic blocks containing the corresponding *GmIAA* genes, which experienced the WGD events. Gene names in red show dispersed duplicates. Color key at the bottom represents row wise *Z*-score.

For soybean, normalized RNA-seq data from 19 tissues were used, which included various tissues/organs, seed compartments, and stages of seed development. *GmIAA* genes showed specific and overlapping expression patterns in various tissues/organs and stages of development analyzed (**Figure 5**, Supplementary Table S7), indicating they might execute specific functions or work redundantly. *GmIAA60* was specifically expressed in WS (whole seedling), while its paralog *GmIAA39* expressed specifically in Ds (dry seed), indicating their functional divergence. *GmIAA15* has higher expression in root (**Figure 5**, Supplementary Table S7) and presence of root-responsive *cis*-regulatory element in its promoter (Supplementary Table S6), suggested its role in root development. In *Arabidopsis*, many *IAA* genes (*AtIAA1*, *AtIAA18*, and *AtIAA28*) were also found to have role in root development (Rogg et al., 2001; Yang et al., 2004; Uehara et al., 2008). In addition, paralogs *GmIAA45* and *51* showed higher transcript accumulation in shoot (**Figure 5**, Supplementary Table S7), signifying their functional conservation with role in shoot development, which is further revealed by presence of shoot responsive *cis*-regulatory element in their promoter sequences (Supplementary Table S6). However, *GmIAA6*, *31*, and *33* exhibited higher transcript levels in floral bud (FB) (**Figure 5**, Supplementary Table S7) and detection of pollen specific *cis*-regulatory elements in their promoter sequences suggests their putative role in development of FBs. Many *GmIAA* genes were detected with increased transcript accumulation at various stages/organs of seed development too. For instance, *GmIAA49* exhibiting specific expression in Gs (globular stage seed), *GmIAA13, 35, 43, 54*, and *56* in GloEP (globular stage embryo proper), *GmIAA5* in GloS (globular stage suspensor), *GmIAA22* and *GmIAA23* in Cs (cotyledon stage seed), *GmIAA59* in EmSCP (early maturation seed coat parenchyma), *GmIAA62* in EmEA (early maturation embryonic axis), and *GmIAA42* in CoL (late maturation cotyledon) (**Figure 5**, Supplementary Table S7). In addition, many seed related *cis*-regulatory elements were detected in their promoter sequences, such as S000143, S000353, S000449, S000148, S000421, S000292, S000144, S000419, S000420, S000100, S000102, and S000377 (Supplementary Table S6), that further added support for their putative role in seed development. In *Arabidopsis*, two Aux/IAA proteins have also been reported for their involvement in seed development (Hamann et al., 1999; Ploense et al., 2009). In the gain-of-function mutant, *iaa18*, PIN1 was asymmetrically expressed with stronger expression at only one side of the embryo and caused aberrant cotyledon outgrowth in the embryos (Ploense et al., 2009). Another, gain-of-function mutant, *iaa12/bdl*, also showed cotyledonary defects (Hamann et al., 1999). Most of *GmIAA* genes showed relative low expression levels in soybean CoS (seedling cotyledon), L (trifoliate leave), Hs (heart stage seed), Es (early maturation stage seed), EcoEM (early maturation embryonic cotyledon), and CoM (mid-maturation cotyledon) tissues (**Figure 5**, Supplementary Table S7).

On the whole, the tissue-preferential expression exhibited by several *Aux/IAA* genes in chickpea and soybean is indicative of their involvement in biology of specific plant tissues and developmental processes. It would be interesting to further validate their functions in transgenics.

### Overlapping and Differential Expression Patterns of Duplicated *GmIAA* Genes

Duplicated genes possibly lead to subfunctionalization (separation of original function), neofunctionalization (gain of novel function), or nonfunctionalization (loss of original function) based on their evolutionary fates (Prince and Pickett, 2002). Therefore, we also examined the functional redundancy of duplicated *GmIAA* genes. In soybean, 50% of the paralogs from the recent WGD event were found to be differentially expressed and thus might have undergone functional divergence. Among *GmIAAs*, 16 paralog pairs (*GmIAA1/30, GmIAA3/28, GmIAA6/33, GmIAA8/32, GmIAA9/48, GmIAA411/56*, *GmIAA13/58, GmIAA17/24, GmIAA34/41, GmIAA36/63, GmIAA37/62, GmI AA38/61, GmIAA40/53*, *GmIAA43/54*, *GmIAA44/52*, and *GmIAA45/51*) representing segmental duplications shared almost indistinguishable expression patterns (**Figure 5**, Supplementary Table S7). On the contrary, the expression patterns of another eight paralogs (*GmIAA10/55, GmIAA14/59, GmIAA15/18, GmIAA16/19, GmIAA21/26, GmIAA22/27, GmIAA39/60*, and *GmIAA46/50)* diversified significantly (**Figure 5**). Interestingly, paralogs from much earlier duplication events (legume WGD and gamma WGT) have more diverged expression patterns. For example, three paralog gene pairs of B2 subfamily, *GmIAA5/35, GmIAA13/58*, and *GmIAA40/53* diverged into two clades after gamma WGT event. After experiencing WGT, *GmIAA40/53* formed one paralog pair, and other was composed of *GmIAA5/35* and *GmIAA13/58* (**Figure 5**). The former paralog gene pair was expressed during the stages of seed development, but the two latter paralog gene pairs detached after the legume WGD event, were highly expressed in the GloEP (**Figure 5**, Supplementary Table S7). Other paralog genes from different divergence events also showed similar expression divergence (**Figure 5**). Besides gamma WGT, legume and *Glycine* WGD also contributed to the expression divergence of paralog *GmIAA* genes. For instance, paralog genes, *GmIAA6/33* exhibited higher expression in stem and FB, whereas *GmIAA11/56* revealed higher expression only in stem (**Figure 5**, Supplementary Table S7). Similarly, paralog genes *GmIAA22/27*, separated from *Glycine* WGD showed distinctively higher expression in Cs (cotyledon stage seed) and CoS (seedling cotyledon), respectively (**Figure 5**, Supplementary Table S7). These results indicate that gamma, legume and *Glycine* WGT events contributed significantly in functional diversity of *GmIAA* gene paralogs.

Altogether, we can speculate that *GmIAAs* have been retained by significant subfunctionalization in soybean during the course of evolution. Meanwhile, it is interesting to note that most of the paralog genes with similar expression profiles belong to the same subfamily and grouped as sister pairs in the phylogenetic tree (**Figures 1** and **5**). For example, two paralogs, *GmIAA13/58* and *GmIAA5/35* in the same subfamily formed sister pairs and displayed similar expression patterns (**Figure 5**). The similar expression pattern of genes from same subfamily of phylogenetic tree indicates that most of these genes may have evolved coordinately in coding and regulatory (promoter) regions, leading to their functional redundancy. Such functional redundancy has been reported in *Aux/IAA* family in *Arabidopsis* too (Overvoorde et al., 2005).

### Differential Expression Patterns of *Aux/IAA* Genes under Abiotic Stress

**FIGURE 6:**
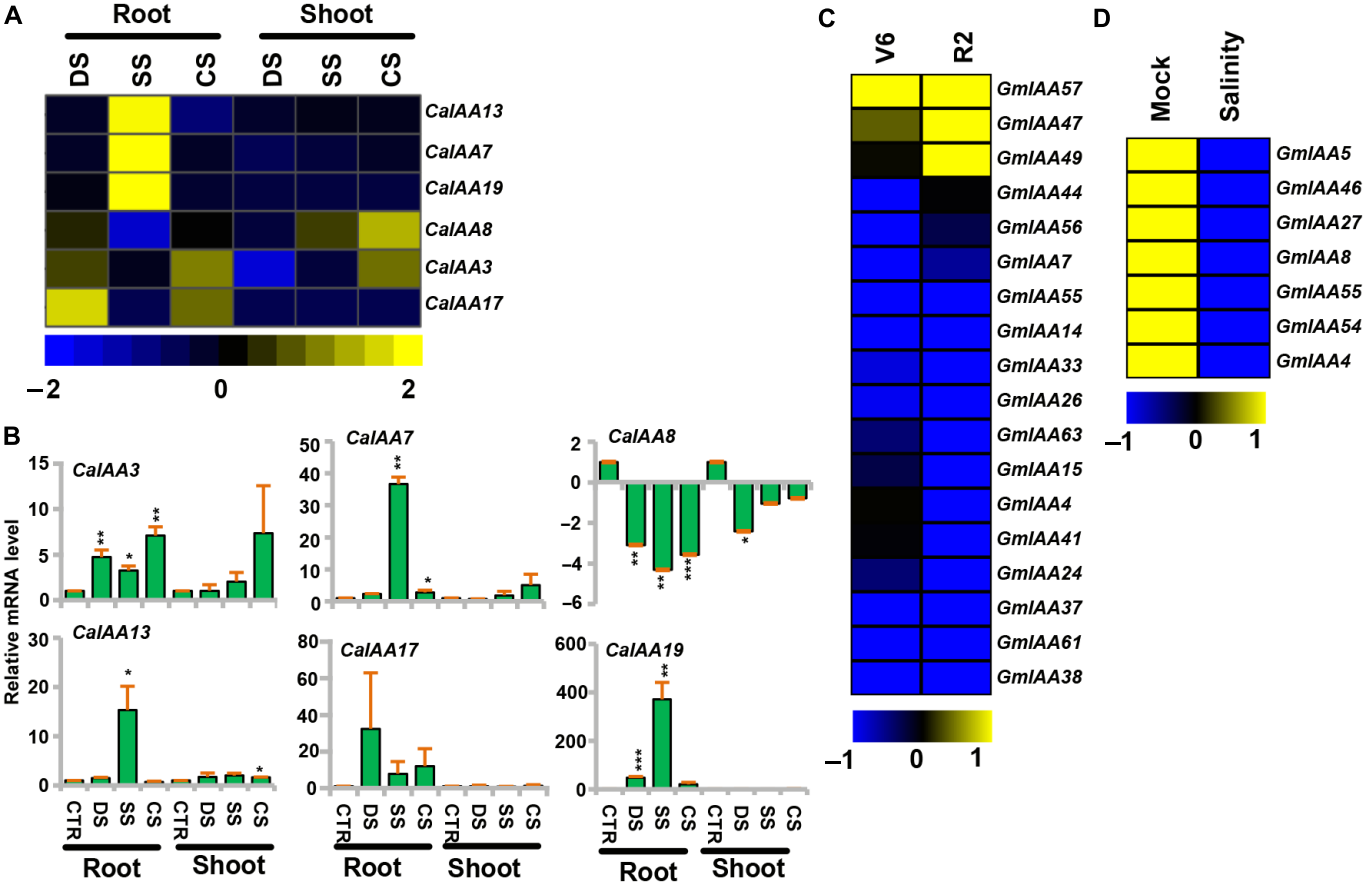
**Expression profiles of *CaIAA* and *GmIAA* genes under abiotic stress conditions**. **(A)** Heatmap shows differential expression of *CaIAA* genes based on RNA-seq data. **(B)** qRT–PCR analysis of *CaIAA* genes under various stress treatments. Root and shoot control (CTR) was taken as a reference to determine relative mRNA level under stress conditions. Error bars indicate standard error of mean. Data points marked with asterisk (^∗^*P* ≤ 0.05, ^∗∗^*P* ≤ 0.01, and ^∗∗∗^*P* ≤ 0.001) indicate statistically significant difference between control and stress treatments. **(C,D)** Differential expression of *GmIAA* genes in response to drought and salinity stress conditions. Color scale shows log_2_ fold change relative to control sample. DS (desiccation), SS (salinity), CS (cold stress), V6 (vegetative stage leaves), R2 (reproductive stage leaves).

Plants are frequently exposed to environmental stresses, like desiccation, salinity, and cold during their life cycle, which affect their growth and development. Several reports highlighted that the auxin-responsive genes were also engaged in various stress responses (Ghanashyam and Jain, 2009; Jain and Khurana, 2009; Wang et al., 2010a; Kumar et al., 2012; Cakir et al., 2013). To gain more insights into the role of chickpea and soybean *Aux/IAA* genes in abiotic stress tolerance, we analyzed their expression profiles under desiccation, salinity, and cold stresses using RNA-seq data for chickpea (Garg et al., 2015) and microarray data for soybean. Many of the chickpea and soybean *Aux/IAA* genes showed induction under desiccation, salinity and/or cold stresses (**Figure 6**). For instance, transcript level of *CaIAA3* was induced significantly under desiccation in root, whereas it was induced in both root and shoot under cold (**Figures 6A,B**, Supplementary Table S8) and its promoter sequence harbors desiccation and cold responsive *cis*-regulatory element (S000407; Supplementary Table S6), indicating its role in desiccation and cold stress. *CaIAA7* showed induction under salinity in root and in shoot under cold stress, while *CaIAA13* was up-regulated in root under salinity (**Figures 6A,B**, Supplementary Table S8). Transcript level of *CaIAA17* was found to be markedly induced in root under desiccation and cold stresses (**Figures 6A,B**, Supplementary Table S8), indicating its role in desiccation and cold stress responses. However, *CaIAA19* illustrated enhanced expression in root under both desiccation and salt stresses (**Figures 6A,B**, Supplementary Table S8), signifying its role in root under abiotic stresses. In rice, *OsIAA9* and *OsIAA20* have been found to be induced under both desiccation and salinity stress conditions (Jain and Khurana, 2009). Further, putative salt stress-related *cis*-element (S000453) was found in promoters of *CaIAA3*, *7*, *13*, and *19* (Supplementary Table S6), which has been demonstrated to be responsible for salt stress response (Park et al., 2004). In response to desiccation and salt stresses, the transcript level of *CaIAA8* was suppressed in shoot and root (**Figures 6A,B**, Supplementary Table S8), respectively, indicating that the function of this gene is related to desiccation and salt stresses. Many *SbIAA* genes of *Sorghum bicolor* have been found down-regulated under drought conditions (Wang et al., 2010a). All the differentially expressed *CaIAAs* were analyzed through qRT-PCR also and expression patterns obtained from qRT-PCR and RNA-seq were correlated well (**Figures 6A,B**).

In soybean, *GmIAA57* revealed distinctly higher transcript accumulation in vegetative stage (V6) leaves under drought stress, while paralogous pair *GmIAA47* and *49* showed noticeably increased accumulation of transcripts in reproductive stage (R2) leaves under drought stress (**Figure 6C**, Supplementary Table S8). Their promoter sequences showed presence of desiccation responsive *cis*-regulatory elements (S000174, S000413; Supplementary Table S6), indicating their function in drought stress responses. In response to salt stress, the transcript levels of *GmIAA4*, *5*, *8*, *27*, *46*, *54*, and *55* were decreased in seedling (**Figure 6D**, Supplementary Table S8), demonstrating the function of these genes related to salt stress. Although the salt stress-related *cis*-element (S000453) was found in the promoters of *GmIAA4*, *5*, *8*, *27*, *46*, *54*, and *55* (Supplementary Table S4), which is reported to induce the transcript level under salt stress (Park et al., 2004), their expression levels were significantly down-regulated under salt stress (**Figure 6D**, Supplementary Table S8). This might indicate that some unidentified *cis*-regulated elements may play an important role in regulating the expression of these *GmIAAs* during stress responses in soybean. Moreover, consistent with our result, many *OsIAA* genes (*OsIAA7*, *8*, *12*, *14*, *17*, *21*, *25*, and *31*) have also been reported to be suppressed in rice under salt stress (Song et al., 2009). The present study clearly revealed that the many of the *Aux/IAA* genes from chickpea and soybean were expressed at significantly higher levels under drought, cold, and salt treatments. It will be interesting to further investigate them to understand their role in abiotic stresses response/signaling.

## Conclusion

In this study, we have performed a comprehensive analysis of *Aux/IAA* genes in chickpea and soybean and provided insights on the evolution of this gene family. The comprehensive expression profiling indicated that members of *Aux/IAA* gene family are involved in many plant responses during development and abiotic stress conditions. Particularly, *CaIAA1*, *3*, *4*, *11*, *12*, *13*, *15*, *17*, *18*, and *21* in chickpea and *GmIAA6*, *13*, *22*, *23*, *31*, *33*, *35*, *39*, *42*, *43*, *45*, *51*, *54*, *56*, *60*, and *62* in soybean were found to have role in various aspect of development, including root, stem, flower bud, flower, and seed development. Further, *CaIAA3*, *7*, 8, *13*, and *17* in chickpea and *GmIAA4*, *5*, *8*, *27*, *46*, *47*, *54*, and *55* in soybean revealed their putative function in abiotic stress responses. The presence of important *cis*-regulatory elements related to various development processes and abiotic stress responses in the promoter of these genes also provided insights into their putative function. These genes are important candidates for further functional characterization. Our analysis suggested that the duplicated *Aux/IAA* genes may perform specific function due to their subfunctionalization. Overall, information reported here for the *CaIAAs* and *GmIAAs* genes should facilitate further investigations related to their functions in plant development and stress responses.

## Conflict of Interest Statement

The authors declare that the research was conducted in the absence of any commercial or financial relationships that could be construed as a potential conflict of interest.

## Footnotes

1 http://cicar.comparative-legumes.org/

2 http://nipgr.res.in/CGAP2/home.php

3 http://pfam.sanger.ac.uk/search

4 http://smart.embl-heidelberg.de

5 http://web.expasy.org/compute_pi/

6 http://gsds.cbi.pku.edu.cn

7 http://meme-suite.org/

8 http://chibba.agtec.uga.edu/duplication

9 http://www.soybase.org/soytedb

10 http://www.seedgenenetwork.net/media

11 https://sogo.dna.affrc.go.jp/cgi-bin/sogo.cgi?sid=&lang=ja&pj=640&action=page&page=newplace
