# Supplementary material for: Genome-wide survey and comprehensive expression profiling of Aux/IAA gene family in chickpea and soybean
Source: Front Plant Sci. 2015 Oct 27;6:918. doi: 10.3389/fpls.2015.00918 (PMC4621760; doi:10.3389/fpls.2015.00918)
Supplement: Supplementary file 1 [file Data_Sheet_1.ZIP › Data/162368_Jain_Table_1.PDF]

Supplemental information

Figure S1 Diagram showing sequence similarity of chickpea and soybean *Aux/IAA* genes

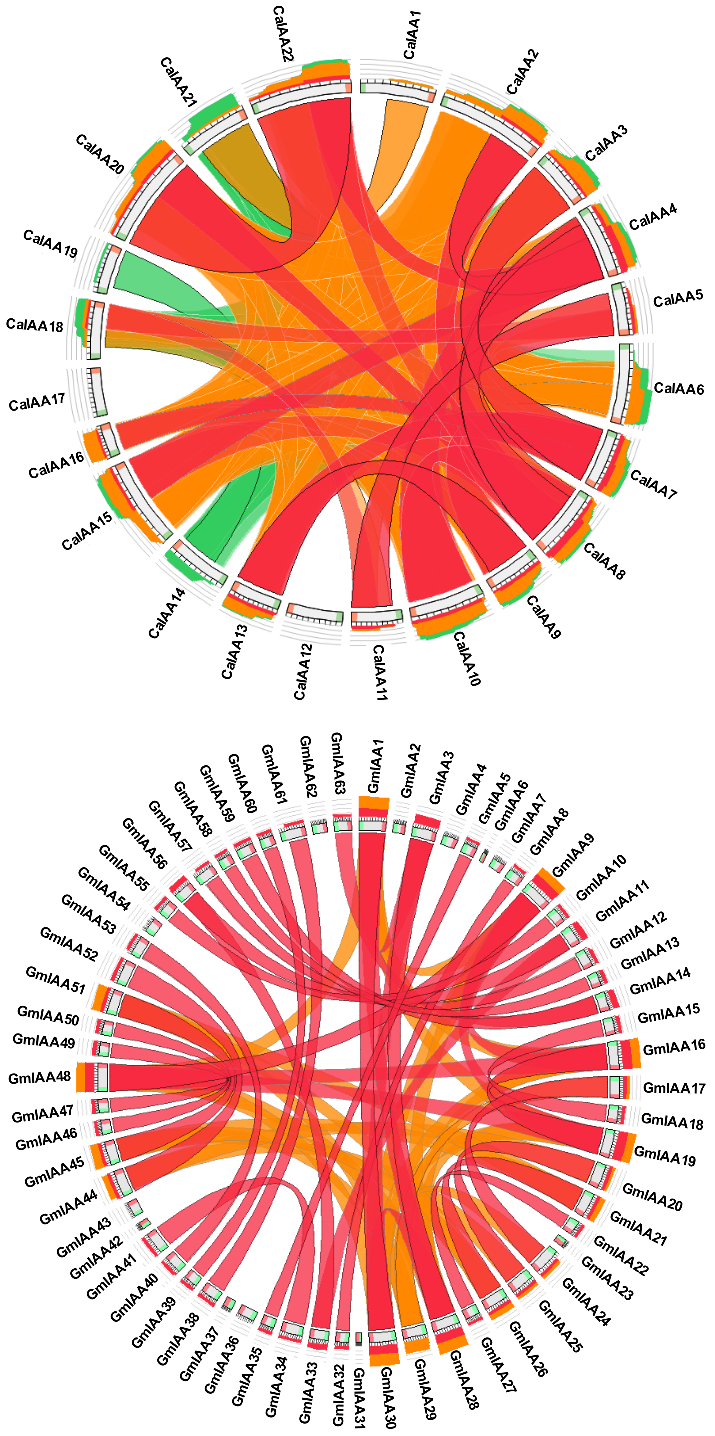

Circoletto tool (http://tools.bat.infspire.org/circoletto/) was used to determine and plot sequence similarity. Red color show highest similarity followed by orange and green colors.

Table S1 Primer sequences of *CaIAA* genes used for qRT-PCR.

Gene name	Primer sequence
CaIAA3	**F-**CAAGGATGGTGATTGGATGCT<br>**R-**CTCTTGCAGGAAGATATGAACATGTT
CaIAA7	**F-**TCTAATACCATATGTCATGACCAAGACA<br>**R-**TGGTGGCCATCCCACTACTT
CaIAA8	**F-**TGATGGATCTTCTTAACAGCTCTGA<br>**R-**GCATCCAGTCGCCATCCTTA
CaIAA13	**F-**AAAAATAGTTTGCAAGAGGCTGAAG<br>**R-**GAGCTCCATCCATGCTCACTTT
CaIAA16	**F-**GTGACTGGATGCTGGTTGGA<br>**R-**AATCCTCAGCCTCTTGCATGA
CaIAA17	**F-**CTTCTACTTGCTGGTGACCTTACTTG<br>**R-**GCCGGAACTATCCGAATTCTC
CaIAA18	**F-**GATGTGCCTTGGGAGATGTTC<br>**R-**CAGCTGTTAGCTCTTGTGATCTTCA
CaIAA19	**F-**CAATCAAAGTTCCACTACCACTATCAC<br>**R-**GCCGACCCACCAAACTTTCT
CaIAA21	**F-**GCAAAGAAGCCACGAAAAGG<br>**R-**TCACCAGGTGGAGCAAGTCTAA
Elongation factor-1 α	**F-** TCCACCACTTGGTCGTTTTG<br>**R-** CTTAATGACACCGACAGCAACAG

Table S3 Colinearity of *GmIAA* genes.

Subfamilies	Gene name	Type	Ks<0.3	0.3<Ks<1.3	Ks>1.3
A1	*GmIAA8*	4	*GmIAA32*	*GmIAA10, GmIAA55*	*GmIAA37, GmIAA62*
	GmIAA32	4T		*GmIAA55*	*GmIAA62*
	GmIAA10	4T	*GmIAA55*	*GmIAA32*	
	GmIAA55	4T		*GmIAA62*	
	GmIAA37	4T	*GmIAA62*		
	GmIAA31	--			
	GmIAA4	1			
A2	*GmIAA22*	4	*GmIAA27*	*GmIAA46, GmIAA50*	
	GmIAA27	4		*GmIAA46, GmIAA50*	
	GmIAA46	4	*GmIAA50*		
	GmIAA47	4	*GmIAA49*		
A3	*GmIAA1*	4	*GmIAA30*	*GmIAA3, GmIAA28*	*GmIAA9, GmIAA16, GmIAA19, GmIAA48*
	GmIAA3	4	*GmIAA28*	*GmIAA30*	
	GmIAA16	4	*GmIAA19*	*GmIAA48*	*GmIAA28, GmIAA30*
	GmIAA9	4	*GmIAA48*	*GmIAA16, GmIAA19*	*GmIAA28, GmIAA30*
	GmIAA19	4		*GmIAA48*	*GmIAA28, GmIAA30*
	GmIAA28	4		*GmIAA30*	
	GmIAA30	4			*GmIAA48*
	GmIAA17	4	*GmIAA24*	*GmIAA29*	*GmIAA45, GmIAA51*
	GmIAA21	4	*GmIAA26*	*GmIAA45, GmIAA51*	*GmIAA24*
	GmIAA24	4		*GmIAA29*	*GmIAA26, GmIAA45, GmIAA51*
	GmIAA26	4		*GmIAA45, GmIAA51*	*GmIAA29*
	GmIAA29	4			*GmIAA29,GmIAA45, mIAA51*
	GmIAA45	4	*GmIAA51*		
	GmIAA2	1			
A4	*GmIAA38*		*GmIAA61*		*GmIAA56*
	GmIAA6	4T	*GmIAA33*	*GmIAA11, GmIAA56*	*GmIAA38, GmIAA61, GmIAA63*
	GmIAA7	3			
	GmIAA33	4T		*GmIAA56*	*GmIAA36, GmIAA38, GmIAA61, GmIAA63*
	GmIAA11	4T	*GmIAA56*	*GmIAA33, GmIAA38*	*GmIAA36,GmIAA63*
	GmIAA56	4T		*GmIAA61*	*GmIAA63*
	GmIAA14	4	*GmIAA59*	*GmIAA36, GmIAA63*	*GmIAA33*
	GmIAA59	4		*GmIAA63*	
	GmIAA36	4		*GmIAA59, GmIAA63*	*GmIAA38, GmIAA56, GmIAA61*
B1	*GmIAA34*	4	*GmIAA41*	*GmIAA57*	
	GmIAA41	4		*GmIAA57*	
	GmIAA12	1			
B2	*GmIAA43*	4	*GmIAA54*		
	GmIAA15	4	*GmIAA18*		*GmIAA54*
	GmIAA18	4			*GmIAA54*
	GmIAA44	4	*GmIAA52*		
	GmIAA20	4	*GmIAA25*	*GmIAA44, GmIAA52*	
	GmIAA25	4		*GmIAA44, GmIAA52*	
	GmIAA40	4	*GmIAA53*		*GmIAA58*
	GmIAA53	4			*GmIAA58*
	GmIAA35	4		*GmIAA58*	*GmIAA40*
	GmIAA5	4	*GmIAA35*	*GmIAA13, GmIAA58*	*GmIAA53*
	GmIAA13	4	*GmIAA58*	*GmIAA35*	*GmIAA40, GmIAA53*
	GmIAA39	4	*GmIAA60*		

Duplication type noted as 0, 1, 2, 3 and 4 stands for singleton, dispersed, proximal, tandem and WGD/segmental, respectively. 4T, tandem genes in collinear blocks.

Table S4 Transposable elements proximal to *GmIAA* genes.

Element name	Class	Chromosome	Strand	Start position	End position	Distance (Kb)
DTH_uuu_Gm1-13	II	Gm01		4258348	4259083	0.19
GmIAA2		Gm01	-	4258161	4256747	
RLC_Gmr5_Gm1-3	I	Gm01		4246497	4248146	8.60
DTH_uuu_Gm2-1	II	Gm02		94092	94479	35.64
GmIAA4		Gm02	-	58453	56569	
RLG_Gmr43_Gm2-1	I	Gm02		52560	53743	2.83
RLC_Gmr456_Gm3-8	I	Gm03		37418089	37419134	28.73
GmIAA10		Gm03	-	37389357	37387584	
DTT_uuu_Gm3-64	II	Gm03		37374562	37374810	12.77
DTT_uuu_Gm3-65	II	Gm03		38080792	38081058	93.35
GmIAA12		Gm03	+	38174406	38178950	
DTH_uuu_Gm3-57	II	Gm03		38233212	38233353	54.26
DTT_uuu_Gm3-64	II	Gm03		37374562	37374810	27.59
GmIAA11		Gm03	+	37402403	37406031	
RLC_Gmr456_Gm3-8	I	Gm03		37418089	37419134	12.06
RLC_Gmr40_Gm7-6	I	Gm07		15989307	15991832	15.11
GmIAA23		Gm07	+	16006946	16011150	
RLC_Gmr6_Gm7-12	I	Gm07		16028722	16029836	17.57
DTH_uuu_Gm10-12	II	Gm10		2829789	2830017	59.45
GmIAA32		Gm10	-	2770339	2768241	
DTM_uuu_Gm10-4	II	Gm10		2709504	2709985	58.26
DTM_uuu_Gm10-4	II	Gm10		2709504	2709985	74.99
GmIAA33		Gm10	+	2784976	2788133	
DTH_uuu_Gm10-12	II	Gm10		2829789	2830017	41.66
DTM_uuu_Gm10-117	II	Gm10		41508578	41518868	171.13
GmIAA37		Gm10	-	41337449	41336339	
DTH_uuu_Gm10-51	II	Gm10		41303337	41303783	32.56
DTH_uuu_Gm10-51	II	Gm10		41303337	41303783	67.78
GmIAA38		Gm10	+	41371565	41374446	
DTM_uuu_Gm10-117	II	Gm10		41508578	41518868	134.13
RLG_Gmr3_Gm13-43	I	Gm13		25309795	25310057	2.69
GmIAA42		Gm13	-	25307109	25305915	
RLG_Gmr27_Gm13-6	I	Gm13		25283427	25283710	22.21
DTH_uuu_Gm15-5	II	Gm15		1075117	1075561	70.79
GmIAA49		Gm15	-	1004331	1002510	
DTM_uuu_Gm15-4	II	Gm15		983218	983854	18.66
DTH_uuu_Gm15-5	II	Gm15		1075117	1075561	58.96
GmIAA50		Gm15	-	1016161	1012651	
DTM_uuu_Gm15-4	II	Gm15		983218	983854	28.80
DTH_uuu_Gm20-63	II	Gm20		44664900	44665619	20.10
GmIAA61		Gm20	-	44644802	44642090	
DTH_uuu_Gm20-62	II	Gm20		44587165	44587600	54.49
DTH_uuu_Gm20-63	II	Gm20		44664900	44665619	19.51
GmIAA62		Gm20	+	44685128	44686751	
RLG_Gmr235_Gm20-8	I	Gm20		44698041	44698594	11.29

Class I, retrotransposon; Class II, DNA transposon. Transposable elements were searched within 200kb upstream and downstream of each target gene at soytedb database (**http://www.soybase.org/soytedb**). Only the nearest upstream and downstream transposable elements are shown. The distances are the length between the nearest transposable element and the target *Aux/IAA* genes.

Table S5 Duplicated gene pairs of *GmIAA* genes with Ka/ Ks values and time of duplication.

Duplicated gene pair		Ka	Ks	Mya (Time)
GmIAA1	GmIAA3	0.08	0.72	55.55
GmIAA1	GmIAA9	0.27	1.75	134.60
GmIAA1	GmIAA16	0.24	1.99	152.89
GmIAA1	GmIAA19	0.25	2.22	170.45
GmIAA1	GmIAA28	0.09	0.79	60.55
GmIAA1	GmIAA30	0.02	0.14	10.40
GmIAA1	GmIAA48	0.27	1.42	109.16
GmIAA3	GmIAA28	0.04	0.16	12.27
GmIAA3	GmIAA30	0.08	0.66	50.58
GmIAA5	GmIAA13	0.13	0.57	43.84
GmIAA5	GmIAA35	0.02	0.11	8.62
GmIAA5	GmIAA53	0.42	3.44	264.29
GmIAA5	GmIAA58	0.15	0.48	36.69
GmIAA6	GmIAA11	0.05	0.44	33.68
GmIAA6	GmIAA33	0.02	0.03	2.35
GmIAA6	GmIAA38	0.07	1.79	137.72
GmIAA6	GmIAA56	0.04	0.39	30.03
GmIAA6	GmIAA61	0.07	1.63	125.35
GmIAA6	GmIAA63	0.08	1.35	103.47
GmIAA7	GmIAA36	0.29	0.00	0.00
GmIAA8	GmIAA10	0.17	0.52	40.05
GmIAA8	GmIAA32	0.04	0.10	7.95
GmIAA8	GmIAA37	0.10	1.46	112.68
GmIAA8	GmIAA55	0.16	0.47	35.78
GmIAA8	GmIAA62	0.24	1.90	146.26
GmIAA9	GmIAA16	0.11	0.62	47.88
GmIAA9	GmIAA19	0.12	0.64	49.24
GmIAA9	GmIAA28	0.23	1.94	149.46
GmIAA9	GmIAA30	0.26	1.63	125.49
GmIAA9	GmIAA48	0.03	0.14	10.65
GmIAA10	GmIAA32	0.19	0.63	48.10
GmIAA10	GmIAA37	0.15	1.10	84.53
GmIAA10	GmIAA55	0.03	0.07	5.75
GmIAA10	GmIAA62	0.27	1.05	80.42
GmIAA11	GmIAA33	0.10	0.55	42.03
GmIAA11	GmIAA38	0.20	1.02	78.44
GmIAA11	GmIAA36	0.38	2.42	186.17
GmIAA11	GmIAA56	0.02	0.11	8.56
GmIAA11	GmIAA63	0.21	2.09	160.98
GmIAA11	GmIAA61	0.24	1.03	78.87
GmIAA13	GmIAA35	0.12	0.53	40.49
GmIAA13	GmIAA40	0.49	2.27	174.31
GmIAA13	GmIAA53	0.48	2.61	200.44
GmIAA13	GmIAA58	0.02	0.13	10.22
GmIAA14	GmIAA36	0.22	1.16	89.38
GmIAA14	GmIAA33	0.21	2.77	212.79
GmIAA14	GmIAA59	0.04	0.14	10.40
GmIAA14	GmIAA63	0.09	1.04	79.75
GmIAA15	GmIAA18	0.03	0.10	7.42
GmIAA15	GmIAA54	0.74	1.92	147.55
GmIAA16	GmIAA19	0.04	0.11	8.52
GmIAA16	GmIAA28	0.24	1.85	141.96
GmIAA16	GmIAA30	0.25	1.76	135.76
GmIAA16	GmIAA48	0.11	0.51	39.35
GmIAA17	GmIAA24	0.01	0.05	3.59
GmIAA17	GmIAA29	0.12	0.51	39.23
GmIAA17	GmIAA45	0.29	1.40	107.35
GmIAA17	GmIAA51	0.28	1.48	114.03
GmIAA18	GmIAA54	0.71	4.26	327.89
GmIAA19	GmIAA28	0.25	1.72	131.98
GmIAA19	GmIAA30	0.25	1.80	138.27
GmIAA19	GmIAA48	0.12	0.51	39.25
GmIAA20	GmIAA25	0.03	0.05	3.66
GmIAA20	GmIAA44	0.26	0.77	59.18
GmIAA20	GmIAA52	0.23	0.72	55.71
GmIAA21	GmIAA26	0.04	0.12	8.88
GmIAA21	GmIAA24	0.56	2.06	158.25
GmIAA21	GmIAA45	0.24	0.47	35.99
GmIAA21	GmIAA51	0.24	0.53	40.82
GmIAA22	GmIAA27	0.01	0.17	12.77
GmIAA22	GmIAA46	0.11	0.70	53.49
GmIAA22	GmIAA50	0.12	0.65	50.34
GmIAA24	GmIAA26	0.49	1.62	124.43
GmIAA24	GmIAA29	0.19	0.55	42.08
GmIAA24	GmIAA45	0.30	1.47	112.78
GmIAA24	GmIAA51	0.47	1.65	126.66
GmIAA25	GmIAA44	0.27	0.68	52.11
GmIAA25	GmIAA52	0.23	0.69	52.84
GmIAA26	GmIAA29	0.43	1.63	125.33
GmIAA26	GmIAA45	0.23	0.50	38.21
GmIAA26	GmIAA51	0.24	0.54	41.25
GmIAA27	GmIAA46	0.13	0.69	53.15
GmIAA27	GmIAA50	0.12	0.67	51.75
GmIAA28	GmIAA30	0.10	0.69	53.04
GmIAA29	GmIAA45	0.30	1.29	99.45
GmIAA29	GmIAA51	0.33	1.57	121.02
GmIAA30	GmIAA48	0.26	1.48	113.92
GmIAA32	GmIAA37	0.13	0.00	0.00
GmIAA32	GmIAA55	0.17	0.60	45.78
GmIAA32	GmIAA62	0.26	2.46	189.31
GmIAA33	GmIAA36	0.38	2.37	182.55
GmIAA33	GmIAA38	0.20	1.29	99.46
GmIAA33	GmIAA56	0.09	0.59	45.49
GmIAA33	GmIAA59	0.21	0.00	0.00
GmIAA33	GmIAA63	0.19	2.47	190.26
GmIAA33	GmIAA61	0.33	1.35	103.79
GmIAA34	GmIAA41	0.02	0.15	11.38
GmIAA34	GmIAA57	0.33	0.72	55.55
GmIAA35	GmIAA40	0.46	2.03	155.97
GmIAA35	GmIAA58	0.14	0.45	34.28
GmIAA36	GmIAA38	0.33	2.12	162.98
GmIAA36	GmIAA59	0.25	1.21	93.34
GmIAA36	GmIAA56	0.42	3.15	242.27
GmIAA36	GmIAA63	0.15	0.36	27.45
GmIAA36	GmIAA61	0.36	1.74	133.82
GmIAA37	GmIAA55	0.15	1.11	85.62
GmIAA37	GmIAA62	0.03	0.09	6.99
GmIAA38	GmIAA56	0.21	1.35	103.95
GmIAA38	GmIAA61	0.02	0.14	10.69
GmIAA39	GmIAA60	0.02	0.06	4.40
GmIAA40	GmIAA53	0.04	0.13	9.62
GmIAA40	GmIAA58	0.47	2.33	179.12
GmIAA41	GmIAA57	0.30	0.69	53.08
GmIAA43	GmIAA54	0.09	0.15	11.72
GmIAA44	GmIAA52	0.01	0.12	9.04
GmIAA45	GmIAA51	0.02	0.11	8.19
GmIAA46	GmIAA50	0.04	0.11	8.64
GmIAA47	GmIAA49	0.05	0.09	6.64
GmIAA53	GmIAA58	0.46	2.46	188.88
GmIAA55	GmIAA62	0.26	1.10	84.53
GmIAA56	GmIAA63	0.23	2.76	212.22
GmIAA56	GmIAA61	0.23	1.15	88.15
GmIAA59	GmIAA63	0.10	1.13	87.02

Table S6 *Cis*-elements predicted in the promoter (1 kb upstream sequence from the start codon) sequences of *CaIAA* and *GmIAA* genes.

Cis-element	PLACE motif identity	Core motif	*CaIAA gene*	*GmIAA gene*	Biological function
-300CORE	S000001	TGTAAAG	12,14,15,19,20	60,62	Seed, endosperm storage protein
2SSEEDPROTBANAPA	S000143	CAAACAC	1,2,16	9,10,13,20,21,28,29,30,44,55,56	Storage protein, seed
AACACOREOSGLUB1	S000353	AACAAAC	1,3,4,6,10,16,19,20	2,3,7,10,14,15,19,21,25,28,29,40,44,53,55,63	endosperm-specific expression
AGCBOXNPGLB	S000232	AGCCGCC	11,16		Stress signal response, ehylene
ARFAT	S000270	TGTCTC	1,5,6,7,8,16,17,18	1,3,9,13,14,15,16,18,19,22,27,28,30,34,35,37,38,39,44,49,58,60,61	Auxin response element (AuxRE1)
ARR1AT	S000454	NGATT	1,2,3,4,5,6,7,8,9,,10,11,12,13,14,15,16,17,18,19,20,21,22	1,2,3,4,5,6,7,8,9,10,11,12,13,14,15,16,17,18,19,20,21,22,23,24,25,26,27,28,29,30,31,32,33,34,35,36,37,38,39,40,41,43,44,45,46,47,48,49,50,51,52,53,54,55,56,57,58,59,60,61,62,63	Cytokinin response regulator
ARR1AT	S000462	CTCTT	1,2,3,4,5,6,7,8,9,,10,11,13,14,15,16,18,19,20,21,22	1,2,4,6,7,11,12,13,14,15,16,17,18,19,20,21,22,23,24,25,26,27,28,29,30,31,34,35,37,38,39,40,43,44,45,46,47,48,49,50,51,52,53,54,56,57,58,60,62,63	Nodule
AUXREPSIAA4	S000026	KGTCCCAT	3,7,13,16	23,54,62	Auxin response element, root tip meristem
CAATBOX1	S000028	CAAT	1,2,3,4,5,6,7,8,9,,10,11,12,13,14,15,16,17,18,19,20,21,22	1,2,3,4,5,6,7,8,9,10,11,12,13,14,15,16,17,18,19,20,21,22,23,24,25,26,27,28,29,30,31,32,33,34,35,36,37,38,39,40,41,43,44,45,46,47,48,49,50,51,52,53,54,55,56,57,58,59,60,61,62,63	seed
CACTFTPPCA1	S000449	YACT	1,2,3,4,5,6,7,8,9,,10,11,12,13,14,15,16,17,18,19,20,21,22	1,2,3,4,5,6,7,8,9,10,11,12,13,14,15,16,17,18,19,20,21,22,23,24,25,26,27,28,29,30,31,32,33,34,35,36,37,38,39,40,41,43,44,45,46,47,48,49,50,51,52,53,54,55,56,57,58,59,60,61,62,63	Mesophyll-specific expression
CANBNNAPA	S000148	CNAACAC	1,2,5,9,,13,16,22	3,9,10,12,13,20,21,22,28,29,30,34,40,43,44,49,52,54,55,56	Storage protein, embryo-and endosperm
CAREOSREP1	S000421	CAACTC	5,11,18,20	3,8,14,18,22,25,34,35,36,40,41,43,46,47,50,52,53,60	Seed
CATATGGMSAUR	S000370	CATATG	4,5,20,21	7,11,12,14,16,23,33,34,35,37,39,44,50,58,61,62	Auxin-responsiveness
CIACADIANLELHC	S000252	CAANNNNATC	1,4,5,6,7,8,9,,12,14,16,18,21	2,3,4,6,7,8,13,14,15,18,20,23,27,30,32,36,38,39,40,41,43,46,48,49,50,51,52,53,54,58,60,61,63	Circadian expression, shoot, leaf
DOFCOREZM	S000265	AAAG	1,2,3,4,5,6,7,8,9,,10,11,12,13,14,15,16,17,18,19,20,21,22	1,2,3,4,5,6,7,8,9,10,11,12,13,14,15,16,17,18,19,20,21,22,23,24,25,26,27,28,29,30,31,32,33,34,35,36,37,38,39,40,41,43,44,45,46,47,48,49,50,51,52,53,54,55,56,57,58,59,60,61,62,63	Leaf, shoot, endosperm
DPBFCOREDCDC3	S000292	ACACNNG	2,4,7,8,9,,11,13,14,16,17	1,4,5,6,9,10,11,12,13,14,15,16,17,18,19,20,21,24,26,27,30,34,36,37,38,39,43,44,45,46,47,49,50,51,52,53,55,56,57,59,60,62,63	embryo, seed
DRE1COREZMRAB17	S000401	ACCGAGA	3	5,30,32,37,52,60	Drought
DRE2COREZMRAB17	S000402	ACCGAC	7,8,9,,13,16	6,8,10,23,31,32,36,45,46,47,55,62	Drought
DRECRTCOREAT	S000418	RCCGAC	7,8,9,,11,13,14,16	3,6,8,9,10,13,15,18,22,23,31,32,34,36,37,45,46,47,54,55,57,58,60,62	Dehydration
EBOXBNNAPA	S000144	CANNTG	2,3,4,5,6,7,8,9,,10,11,12,13,14,15,16,17,18,19,20,21	1,2,3,4,5,6,7,8,9,10,11,12,13,14,15,16,17,18,19,20,21,23,24,25,26,27,28,29,30,31,32,33,34,35,36,37,38,39,40,41,43,44,45,46,47,48,49,50,51,52,53,54,55,56,57,58,59,60,61,62,63	Storage protein, seed
EMHVCHORD	S000452	TGTAAAGT	14,15,19,20		Endosperm
GARE1OSREP1	S000419	TAACAGA	12,15,19,20	38,51,60	Gibberellin-responive, seed
GARE2OSREP1	S000420	TAACGTA	11,17	26	Gibberellin-responive, seed
GATABOX	S000039	GATA	1,2,3,4,5,6,7,8,9,,10,11,12,13,14,15,16,17,18,19,20,21,22	1,2,3,4,5,6,7,8,9,10,11,12,13,14,15,16,17,18,19,20,21,22,23,24,25,26,27,28,29,30,31,32,33,34,35,36,37,38,39,40,41,43,44,45,46,47,48,49,50,51,52,53,54,55,56,57,58,59,60,61,62,63	Chlorophyll a/b, light regulation, leaf, shoot
GCN4OSGLUB1	S000277	TGAGTCA	6	13,20,24	Endosperm
GT1CONSENSUS	S000198	GRWAAW	1,2,3,4,5,6,7,8,9,,10,11,12,13,14,15,16,17,18,19,20,21,22	1,2,3,4,5,6,7,8,9,10,11,12,13,14,15,16,17,18,19,20,21,22,23,24,25,26,27,28,29,30,31,32,33,34,35,36,37,38,39,40,41,43,44,45,46,47,48,49,50,51,52,53,54,55,56,57,58,59,60,61,62,63	Light regulation, leaf, shoot
GT1GMSCAM4	S000453	GAAAAA	1,2,3,4,5,6,7,9,,11,14,15,16,17,19,21,22	1,2,3,5,7,8,9,10,11,12,13,14,15,16,17,18,19,20,21,22,23,24,25,26,27,28,29,30,32,33,34,35,36,37,38,39,40,41,43,44,45,46,47,48,49,50,51,52,53,54,55,56,57,58,59,60,61,62,63	salt-induced gene expression
GTGANTG10	S000378	GTGA	1,2,3,4,5,6,7,8,9,,10,11,12,13,14,15,16,17,18,20,21,22	1,2,3,4,5,6,7,8,9,10,11,12,13,14,15,16,17,18,19,20,21,22,23,24,25,26,27,28,29,30,31,32,33,34,35,36,37,38,39,40,41,43,44,45,46,47,48,49,50,51,52,53,54,55,56,57,58,59,60,61,62,63	Pollen
IBOXCORENT	S000424	GATAAGR	7,10,12,16	1,16,19,23,36,44,47,59,63	Light
LEAFYATAG	S000432	CCAATGT	2,5,14	1,14,15,26,39,44,49,53	Root apical meristem
LTRE1HVBLT49	S000250	CCGAAA	13,16	3,6,10,11,16,17,19,37,47,49,55,56	low-temperature-responsive elemen, cold
LTREATLTI78	S000157	ACCGACA	7,9,,13	8,32,36,45,47,62	low-temperature-responsive elemen, cold
LTRECOREATCOR15	S000153	CCGAC	7,8,9,,11,12,13,14,15,16,17	3,6,8,9,10,13,14,15,17,18,22,23,27,29,31,32,34,36,37,43,45,46,47,49,52,54,55,57,58,60,62,63	low-temperature-responsive elemen, cold
MYCATERD1	S000413	CATGTG	2,3,7,8,9,,12,13,16,21	3,5,6,9,10,15,18,19,23,26,31,32,36,37,38,39,41,46,47,49,50,51,53,55,56,57,59,60,61,62	Dehydration, water stess
MYCATRD22	S000174	CACATG	2,3,7,8,9,,12,13,16,21	3,5,6,9,10,15,18,19,23,26,31,32,36,37,38,39,41,46,47,49,50,51,53,55,56,57,59,60,61,62	Dehydaration, water stess
MYCCONSENSUSAT	S000407	CANNTG	2,3,4,5,6,7,8,9,,10,11,12,13,14,15,16,17,18,19,20,21	1,2,3,4,5,6,7,8,9,10,11,12,13,14,15,16,17,18,19,20,21,23,24,25,26,27,28,29,30,31,32,33,34,35,36,37,38,39,40,41,43,44,45,46,47,48,49,50,51,52,53,54,55,56,57,58,59,60,61,62,63	Dehydration, Cold, ABA, leaf, seed
NODCON1GM	S000461	AAAGAT	1,2,6,7,9,,11,13,14,15,17,18,20,21	2,3,5,6,7,8,9,10,11,13,14,15,16,17,18,19,21,22,23,24,26,27,28,29,30,31,32,33,35,39,40,41,43,44,45,46,47,50,51,53,54,55,56,59,61,62,63	Nodule
OSE1ROOTNODULE	S000467	AAAGAT	1,2,6,7,9,,11,13,14,15,17,18,20,21	2,3,5,6,7,8,9,10,11,13,14,15,16,17,18,19,21,22,23,24,26,27,28,29,30,31,32,33,35,39,40,41,43,44,45,46,47,50,51,53,54,55,56,59,61,62,63	Root, nodule, arbuscule
OSE2ROOTNODULE	S000468	CTCTT	1,2,3,4,5,6,7,8,9,,10,11,13,14,15,16,18,19,20,21,22	1,2,4,6,7,11,12,13,14,15,16,17,18,19,20,21,22,23,24,25,26,27,28,29,30,31,34,35,37,38,39,40,43,44,45,46,47,48,49,50,51,52,53,54,56,57,58,60,62,63	Root, nodule, arbuscule
POLLEN1LELAT52	S000245	AGAAA	1,2,3,4,5,6,7,8,9,,10,11,12,13,14,15,16,17,18,19,20,21,22	1,2,3,4,5,6,7,8,9,10,11,12,13,14,15,16,17,18,19,20,21,22,23,24,25,26,27,28,29,30,31,32,33,34,35,36,37,38,39,40,41,43,44,45,46,47,48,49,50,51,52,53,54,55,56,57,58,59,60,61,62,63	Pollen specific activation
ROOTMOTIFTAPOX1	S000098	ATATT	1,2,3,4,5,6,7,8,9,,10,11,12,13,14,15,16,17,18,19,20,21,22	1,2,4,5,6,7,8,9,10,11,12,13,14,15,16,17,18,19,20,21,22,23,24,25,26,27,28,29,30,31,32,33,34,35,36,37,38,39,40,41,43,44,45,46,47,48,49,50,51,53,54,55,56,57,58,59,60,61,62,63	Root
RYREPEATLEGUMINBOX	S000100	CATGCAY	4,7,8,12,14,18,19,22	2,5,9,10,11,15,16,18,22,23,25,26,31,32,34,36,40,41,53,55,56,57,58,59	Seed storage protein
RYREPEATVFLEB4	S000102	CATGCATG	8,22	2,9,15,16,22,23,34,56,59	Seed, embryo
SORLIP1AT	S000482	GCCAC	8,11,12,13,14,15,16,20	1,2,3,4,5,7,8,9,12,15,16,17,18,19,20,22,24,27,30,31,32,33,34,36,37,39,41,45,50,51,52,62	phyA, phytochrome, light
SORLIP2AT	S000483	GGGCC		6,10,40,41,47,49,52,53,55,62,63	phyA, phytochrome, light
SORLIP5AT	S000486	GAGTGAG	17	8,10,17,18,39,52,56	phyA, phytochrome, light
SORLREP3AT	S000488	TGTATATAT	4,8,20	13,14,21,26,38,50,58,63	phyA, phytochrome, light
SREATMSD	S000470	TTATCC	2,6,9,	7,10,14,16,17,18,25,26,27,28,29,31,33,36,39,40,41,44,46,47,48,49,50,53,55,59,62,63	sugar-repressive element (SRE)
SURE1STPAT21	S000186	AATAGAAAA	1,11	2,5,43,45,53,63	Sucrose Responsive Element (SURE)
SURE2STPAT21	S000185	AATACTAAT	13,19	12,21,32,38,39,40,45,60,62	Sucrose Responsive Element 2 (SURE2)
TGACGTVMAMY	S000377	TGACGT	14	3,6,31,63	Cotyledons, germinated seeds, seed
WRKY71OS	S000447	TGAC	1,2,3,4,5,6,7,8,9,,10,11,12,13,14,15,16,17,18,19,20,21,22	1,2,3,4,5,6,7,8,9,10,11,12,13,14,15,16,17,18,19,20,21,22,23,24,25,26,27,28,29,30,31,32,33,34,35,36,37,38,39,40,41,43,44,45,46,47,48,49,50,51,52,53,54,55,56,57,58,59,60,61,62,63	Gibberellin signaling pathway repressor, defence response
XYLAT	S000510	ACAAAGAA	6,11,16,18	13,15,20,24,26,32,33,37,41,47,51,53,59,61	Secondary xylem, wood formation
